# Efficacy and Safety of Ocriplasmin Use for Vitreomacular Adhesion and Its Predictive Factors: A Systematic Review and Meta-Analysis

**DOI:** 10.3389/fmed.2021.759311

**Published:** 2022-01-13

**Authors:** Xi Chen, Min Li, Ran You, Wei Wang, Yanling Wang

**Affiliations:** ^1^Department of Ophthalmology, Beijing Friendship Hospital, Capital Medical University, Beijing, China; ^2^Clinical Epidemiology and Evidence-Based Medicine (EBM) Unit, National Clinical Research Center for Digestive Disease, Beijing Friendship Hospital, Capital Medical University, Beijing, China

**Keywords:** ocriplasmin, symptomatic vitreomacular adhesion/vitreomacular traction, macular hole (MH), meta-analysis (as topic), individual participant data analysis

## Abstract

Symptomatic vitreomacular adhesion (sVMA) impedes visual acuity and quality. Ocriplasmin is a recombinant protease, which may be injected into the vitreous cavity to treat this condition, yet controversy remains with respect to its effectiveness and safety, particularly its patient selection standard. In this systematic review, the PubMed, Embase, and the Cochrane Library were searched to identify studies published prior to August 2020 on the impact of ocriplasmin treatment on VMA release, macular hole (MH) closure, and/or related adverse events (AEs). Data were pooled using a random-effects model. Risk ratios (RRs) with 95% CIs were calculated. Of 1,186 articles reviewed, 5 randomized controlled trials and 50 cohort studies were ultimately included, representing 4,159 patients. Ocriplasmin significantly increased the rate of VMA release (RR, 3.61; 95% CI, 1.99–6.53; 28 days after treatment) and MH closure (RR, 3.84; 95% CI, 1.62–9.08; 28 days after treatment) and was associated with visual function improvement. No increased risk for overall AEs was seen in ocriplasmin treatment. The proportion of VMA release and MH closure in patients was 0.50 and 0.36, respectively. VMA release was more likely in patients with absence of epiretinal membrane (ERM). Patients with smaller MH diameter were more likely to achieve MH closure. Evidence from included studies suggests that ocriplasmin is a suitable and safe approach for treating sVMA. ERM and MH status are important factors when considering ocriplasmin treatment.

## Introduction

Symptomatic vitreomacular adhesion (sVMA) typically occurs with incomplete posterior vitreous detachment (PVD) and leads to subsequent loss or distortion of vision (1–3). sVMA can further result in the occurrence of vitreomacular traction (VMT), often coinciding with macular hole (MH) and epiretinal membrane (ERM).

Based on its etiology, treatment of sVMA requires the release of vitreous body traction on the retina. The current standard management option for treating these adhesions is pars plana vitrectomy (PPV), which involves removing the vitreous surgically (4, 5). However, even small-gauge procedure PPV can lead to serious complications including retinal detachment, retinal tears, endophthalmitis, and postoperative cataract formation. A biological agent for non-invasive treatment of VMA known as ocriplasmin (Jetrea; ThromboGenics NV, Leuven, Belgium, UK) was approved as the first drug of its kind by the US Food and Drug Administration on October 17, 2012 (6, 7). Ocriplasmin is composed of the catalytic domain of human plasmin with proteolytic activity against protein components of the vitreous body and vitreoretinal interface. It dissolves the protein matrix responsible for VMA. The approval of ocriplasmin for clinical use was based on the MIVI-TRUST study (8). Since then, randomized controlled trials (RCTs) including MIVI-IIT and OASIS (9, 10), prospective cohort studies, and observational studies including INJECT, ORBIT, and OVIID-1 (11–13) have analyzed the efficacy of and adverse reactions to ocriplasmin. Resulting data show that non-surgical induction of PVD using ocriplasmin can offer the benefits of VMA release and MH closure while eliminating the risks associated with a surgical procedure.

Subgroup analyses on pharmacologic VMA resolution showed that subjects with certain baseline characteristics had higher VMA resolution rate included absence of ERM, presence of MH, small adhesion diameter, phakic lens status, gender, and age (11, 13, 14). Meta-analysis of Jackson et al. further demonstrated that presence of ERM and broad VMA, increasing age, and male gender were associated with decreased treatment response in RCT reports (15).

This study includes a complete search for existing data in this meta-analysis to evaluate the efficacy and safety profile of ocriplasmin for the treatment of sVMA with/without MH, across subgroups defined by the presence of ERM and MH, and also to identify factors which may affect the effectiveness of ocriplasmin including MH diameter, age, gender, and others. Based on our findings, we proposed the optimal profile of patient for treatment with ocriplasmin.

## Materials and Methods

This study is fully compliant with the Preferred Reporting Items for Systematic Reviews and Meta-analyses (PRISMA) statement (16). This study was registered with the International Prospective Register of Systematic Reviews (PROSPERO) (CRD42021228893).

### Data Sources and Search Strategy

The PubMed, Embase, and the Cochrane Library were searched from inception to August 1, 2020. In addition, we checked the websites of the Association for Research in Vision and Ophthalmology (https://www.arvo.org) and the European Society of Ophthalmology (https://soevision.org/organisation) for annual conference abstracts published from inception to August 1, 2020 and the reference lists of all the relevant articles to identify additional studies. Full details of the search strategy and results are given in Supplementary File 1.

### Study Selection

Randomized controlled trials and cohort studies were eligible for inclusion, if they met the following criteria: (1) participants were patients diagnosed with VMA and/or MH and (2) the effectiveness of ocriplasmin on VMA release, MH closure, or vision improvement was reported. For papers reporting data from the same participants with common authors, research centers, and overlapping enrollment periods, the most comprehensive of these was included. Reviews, editorials, letters, guidelines, and protocols as well as articles describing studies with fewer than 10 participants or focused on basic research were excluded.

### Data Extraction and Quality Assessment

Two investigators (XC and ML) independently assessed the eligibility of studies and extracted data in duplicate. Any disagreement on study inclusion or interpretation of data was resolved by consulting the senior investigator (YW). The extracted data included study information (first author, publication year, sample size, region of study, and study design), characteristics of participants (age and gender), treatment details (dose), and disease characteristics (definition of cases, presence of ERM, diameter of VMA, and size of MH).

Study quality was assessed using the Cochrane Collaboration risk of bias tool for RCTs and a published quality appraisal checklist for cohort studies (17). This checklist examines the main domains including study design, population, intervention, outcome measures, statistical analysis, results/conclusions, competing interests, and sources of financial support.

### Statistical Analysis

Characteristics of included studies were described. Heterogeneity between studies was quantified by the *I*^2^-test. An *I*^2^ statistic above 50% was considered to indicate substantial heterogeneity. Random-effects models were used for all the meta-analyses due to clinical heterogeneity inherent in the data. In case of zero event appeared in included studies, 0.5 was added to the event number, as Haldane–Anscombe correction referred.

Pooled risk ratios (RRs) with 95% CIs were calculated to estimate the impact of ocriplasmin vs. placebo/sham for participants with VMA and/or MH in increasing the rate of VMA release, MH closure, vision improvement, associated vitrectomy, or adverse events (AEs). Pooled proportions of eyes with VMA resolution and MH closure after ocriplasmin injection were calculated. Subgroup analyses were performed to examine whether the rate of VMA resolution after ocriplasmin injection was modified by preplanned variables including wet age-related macular degeneration (wAMD), diabetic retinopathy (DR), and retinal vein occlusion (RVO). Further, to reveal the factors associated with VMA release/MH closure, pooled mean difference/odds ratios (ORs) with 95% CIs for each potential factor were estimated as appropriate. The Begg's and Egger's tests and a funnel plot were used to evaluate publication bias.

All the data from included studies whose authors had provided the raw data were included in the individual participant data analysis (IPD) analysis. The receiver operating characteristic (ROC) curves were plotted and the area under the ROCs (AUROCs) were calculated to determine the predict ability of characteristics of participants including age, gender, VMA diameter, and ERM formation for VMA release after ocriplasmin injection. Those characteristics were included in the multivariable logistic regression models and a final model selection was performed using a backward selection process. The maximum Youden index was used to define the optimal cutoff values. Sensitivity and specificity were used to evaluate the predicted performance of each cutoff value.

*P*-values (two-tailed) of < 0.05 were considered as statistically significant. All the analyses were conducted using the meta package of R software, version 3.6.2.

## Results

### Characteristics of the Included Studies

**Figure 1:**
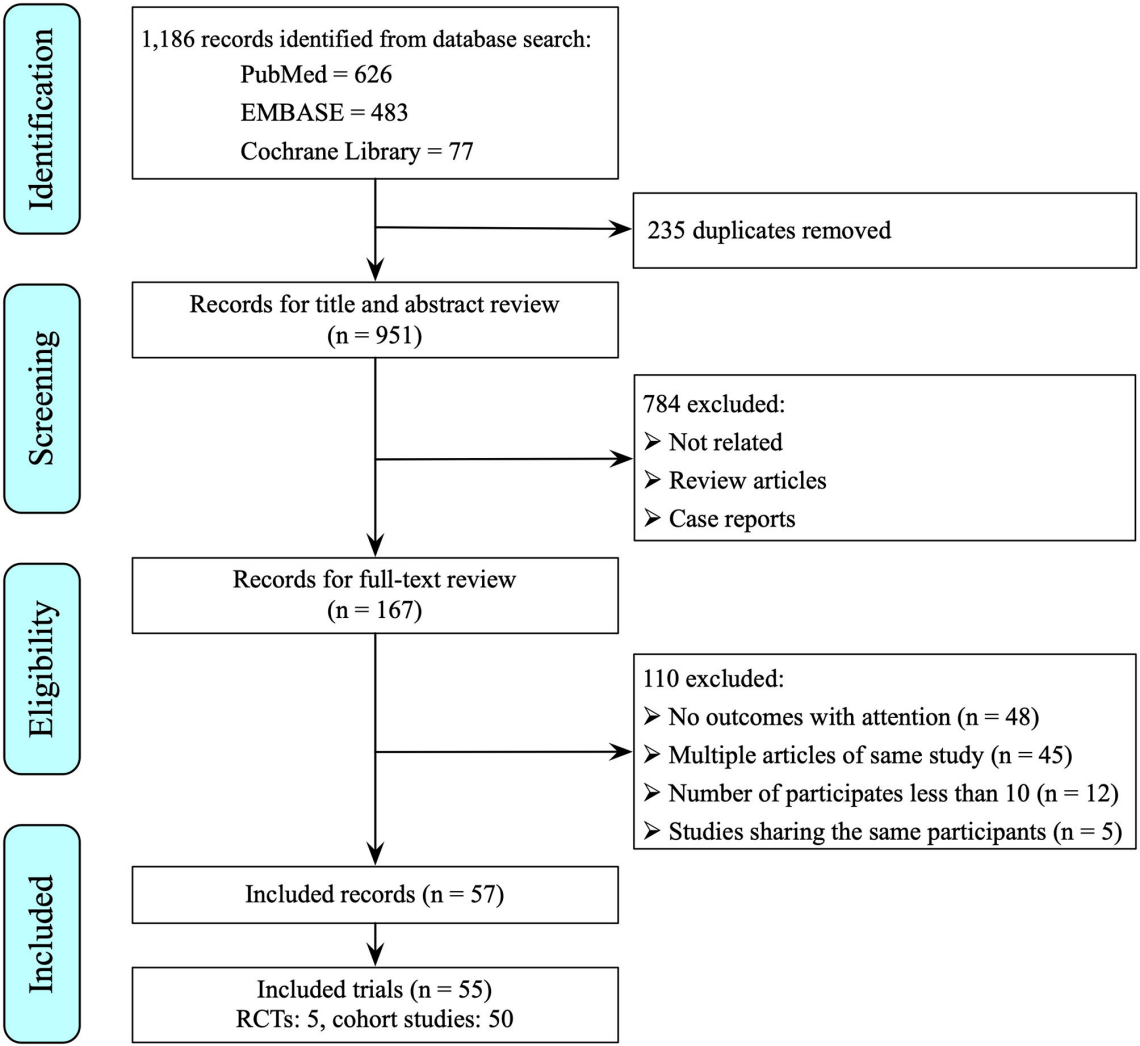
Flow diagram describing the literature screening process.

The search described above yielded 1,186 publications from the PubMed, Embase, and the Cochrane Library databases, of which 235 publications were duplicates. Of the 951 remaining articles, 784 irrelevant articles were identified by reviewing titles and abstracts and were excluded. The full text of the remaining 167 articles were reviewed, after which 110 articles were excluded due to a lack of outcomes with attention, papers reporting data from the same cohort, or participants smaller than 10. Finally, a total of 55 studies (5 RCTs and 50 cohort studies in 57 publishing articles) with 4,159 participants were included in this meta-analysis (Figure 1). All the included studies were conducted in North American and European countries, except one from Australia. The recommended ocriplasmin dose is 125 μg for single intravitreal injection and this was the intervention strategy applied in our included studies (Supplementary Table 1; Supplementary File 2 for full included studies list).

### Therapeutic Effect of Ocriplasmin Injection in RCTs

**Figure 2:**
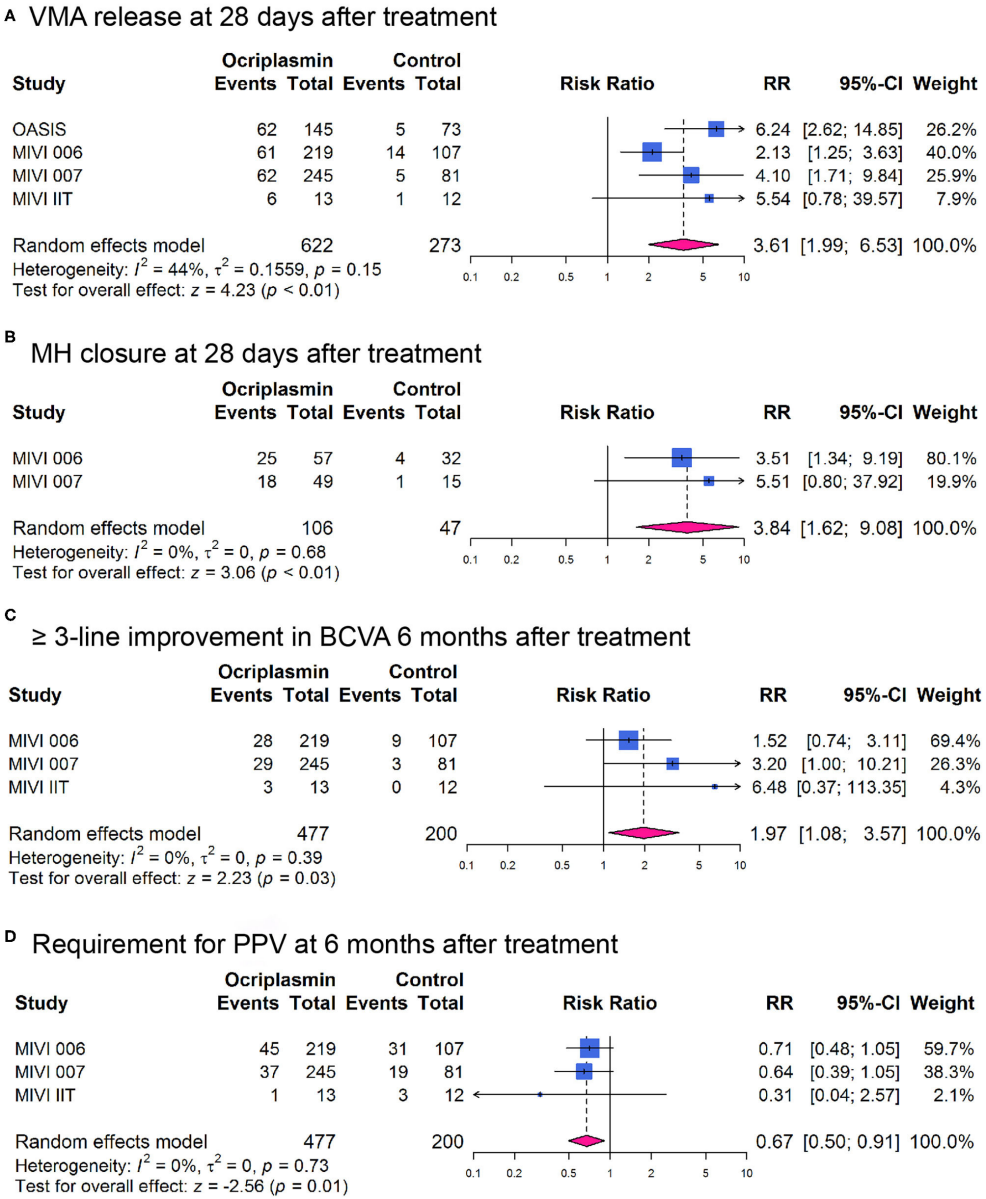
Forest plots of therapeutic effect of ocriplasmin injection compared with controls in included randomized controlled trials. **(A)** Vitreomacular adhesion (VMA) release at 28 days after treatment; **(B)** Macular hole (MH) closure at 28 days after treatment; **(C)** At least 3-line improvement in best corrected visual acuity (BCVA) at 6 months after treatment; **(D)** Incidence of pars plana vitrectomy (PPV) at 6 months after treatment.

Overall, the RR for nonsurgical VMA release was 3.61 [95% CI: 1.99–6.53; *I*^2^ = 44%; *P*_*het*_ = 0.15 (*p*-value for heterogeneity); Figure 2A] in non-wAMD participants at 28 days after treatment, which was higher than reported in wAMD [(18); RR: 2.03; 95% CI: 0.65–6.31]. MH closure was achieved more frequently with ocriplasmin than in the control group (RR 3.84, 95% CI: 1.62–9.08; *I*^2^ = 0%; *P*_*het*_ = 0.68; Figure 2B) at 28 days after treatment, consistent with the OASIS trial that reported the number of participants achieving MH closure at 24 months after ocriplasmin treatment was higher than sham (RR: 1.95; 95% CI: 0.72–5.28).

Best corrected visual acuity (BCVA) improvement of at least three lines at 6 months after treatment was more likely in participants undergoing ocriplasmin treatment than with sham injection (RR: 1.97; 95% CI: 1.08–3.57; *I*^2^ = 0%; *P*_*het*_ = 0.39; Figure 2C). Also, we observed that the OASIS trial reported ≥2-line improvement in BCVA (RR: 1.27; 95% CI: 0.92–1.75). Moreover, comparison of the 25-item National Eye Institute Visual Function Questionnaire-25 (VFQ-25) composite score between ocriplasmin and control treatment data showed that a larger percentage of participants treated with ocriplasmin experienced a ≥5-point (clinically meaningful) improvement in VFQ-25 composite score at 6 months after treatment (RR: 1.33; 95% CI: 1.02–1.73) in MIVI 006 and 007 trials. Accordingly, the percentage of participants with ≥ 5-point worsening was lower with ocriplasmin at 6 months after treatment (RR: 0.62; 95% CI: 0.44–0.86) in MIVI 006 and 007 trials. The OASIS trial reported that the participants receiving ocriplasmin with ≥ 5-point improvement in VFQ-25 composite score at 24 months were also more than control (RR: 1.72; 95% CI: 1.17–2.52) and participants with ≥ 5-point worsening were lower than control (RR: 0.64; 95% CI: 0.31–1.34).

In addition, fewer participants who required PPV were in the ocriplasmin group than were in the sham group at 6 months after treatment (RR: 0.67; 95% CI: 0.50–0.91; *I*^2^ = 0%; *P*_*het*_ = 0.73; Figure 2D), consistent with the OASIS trial that reported the number of participants requiring PPV at 24 months after ocriplasmin treatment was less than control (RR: 0.76; 95% CI: 0.53–1.07).

### Incidence of AEs After Receiving Ocriplasmin Therapy

The proportion of participants experiencing at least one AE was comparable between the ocriplasmin and control groups (RR: 1.13; 95% CI: 0.95–1.34; *I*^2^ = 71%; *P*_*het*_ < 0.01; Supplementary Figure 1A). No significant difference between ocriplasmin and control was found in the incidence of serious AEs (RR: 1.38; 95% CI: 0.44–4.32; *I*^2^ = 64%; *P*_*het*_ = 0.10; Supplementary Figure 1B) and serious ocular AEs (RR: 0.88; 95% CI: 0.58–1.33; *I*^2^ = 12%; *P*_*het*_ = 0.33; Supplementary Figure 1D). It is worth noting, however, that ocular AEs of ocriplasmin therapy were slightly higher than control (RR: 1.20; 95% CI: 1.05–1.37; *I*^2^ = 36%; *P*_*het*_ = 0.18; Supplementary Figure 1C), suggesting that while ocriplasmin therapy did not raise the risk of overall AEs, it may carry a higher risk of ocular AEs.

### Proportion of Ocriplasmin Therapy in Cohort Studies

**Figure 3:**
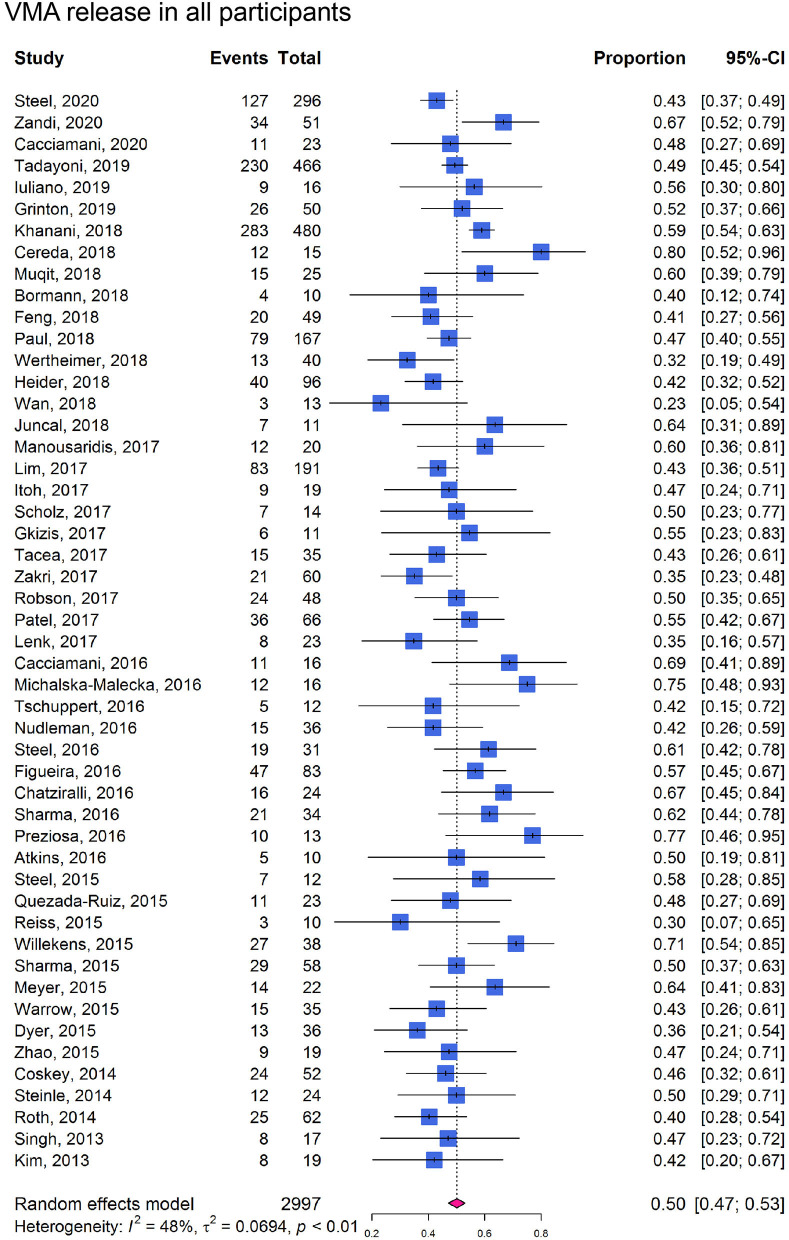
Forest plots of proportion of VMA release in participants receiving ocriplasmin therapy in included cohort studies.

**Figure 4:**
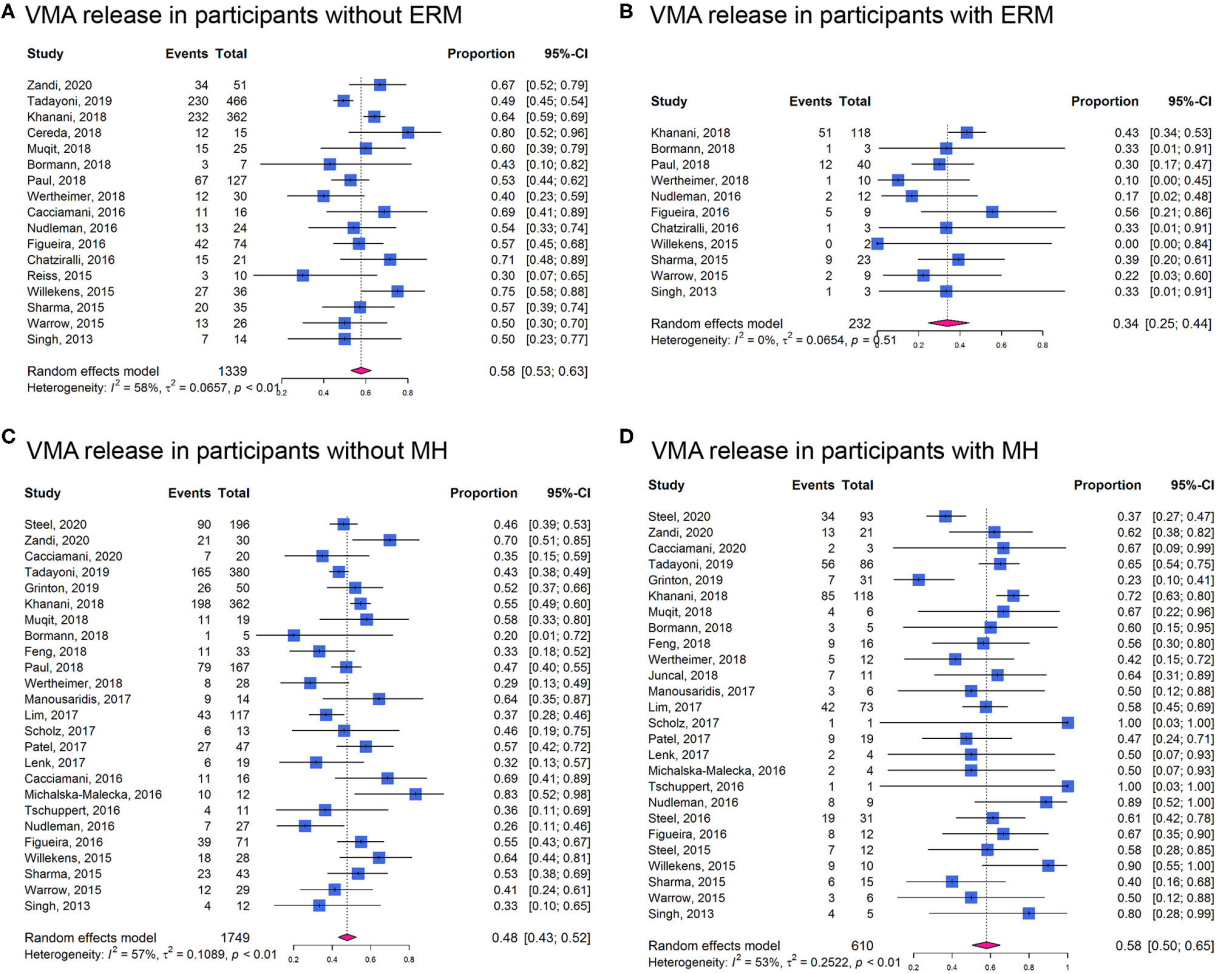
Forest plots of proportion of VMA release in participants receiving ocriplasmin therapy in different groups. **(A)** In participants without epiretinal membrane (ERM); **(B)** In participants with ERM; **(C)** In participants without MH; **(D)** In participants with MH.

In cohort studies, the overall proportion of eyes achieving non-surgical VMA release was 0.50 (95% CI: 0.47–0.53; *I*^2^ = 48%; *P*_*het*_ < 0.01; Figure 3). In participants without ERM at baseline, the proportion of VMA release (0.58, 95% CI: 0.53–0.63; *I*^2^ = 58%; *P*_*het*_ < 0.01; Figure 4A) was higher than those with ERM (0.34, 95% CI: 0.25–0.44; *I*^2^ = 0%; *P*_*het*_ = 0.51; Figure 4B). Participants with MH were more likely to experience VMA release (0.58, 95% CI: 0.50–0.65; *I*^2^ = 53%; *P*_*het*_ < 0.01; Figure 4D) than those without MH (0.48, 95% CI: 0.43–0.52; *I*^2^ = 57%; *P*_*het*_ < 0.01; Figure 4C). Moreover, we found that the proportion of VMA release in participants with or without ERM potentially increased with time, especially in ERM participants after 6 months (Supplementary Figure 2).

**Figure 5:**
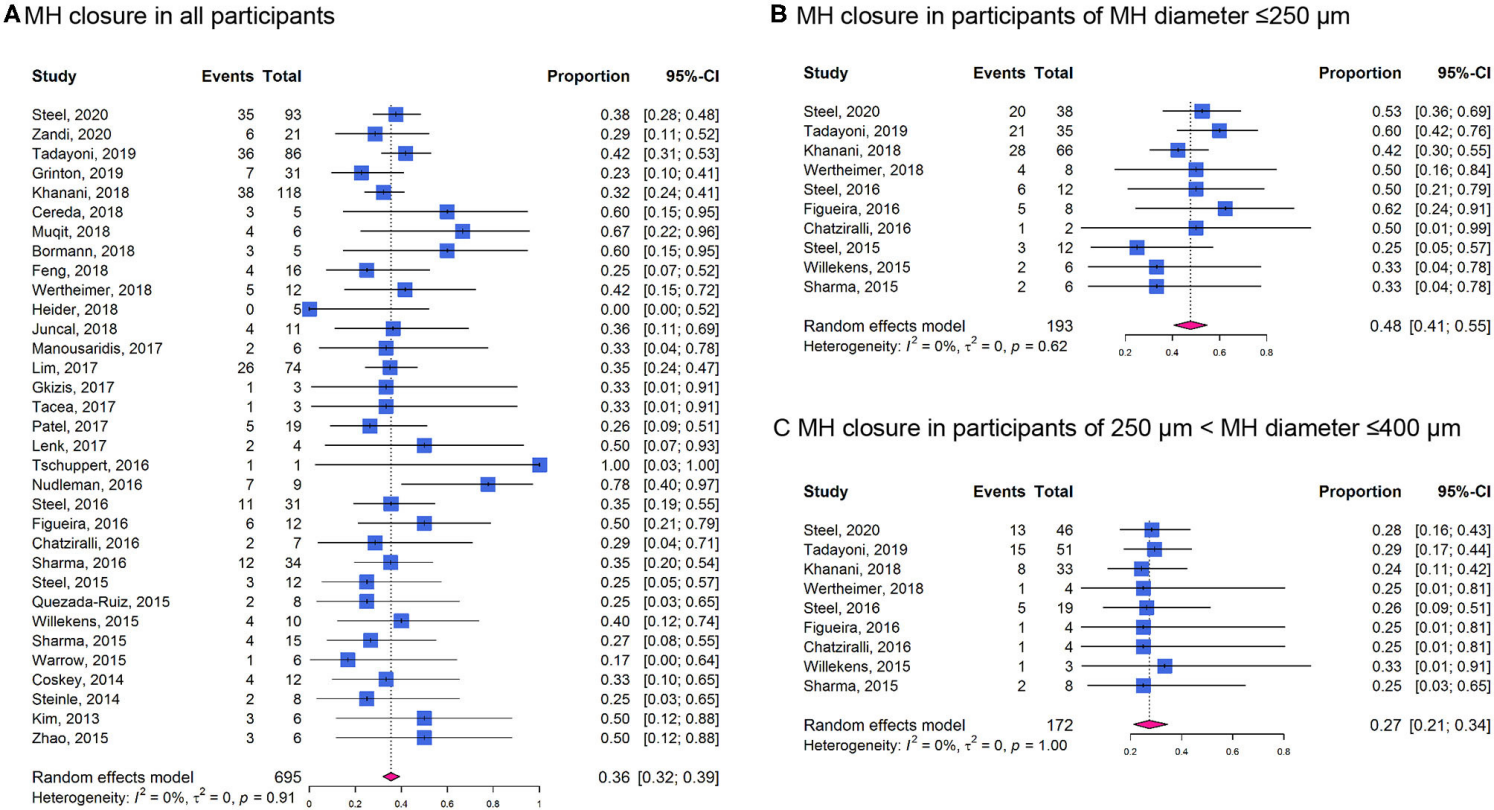
Forest plots of proportion of MH closure in participants receiving ocriplasmin therapy in included cohort studies. **(A)** In all the participants; **(B)** In participants of MH diameter ≤ 250 μm; **(C)** In participants of MH diameter of 250–400 μm.

The overall proportion for MH closure was 0.36 (95% CI: 0.32–0.39; *I*^2^ = 0%; *P*_*het*_ = 0.91; Figure 5A). The proportion in participants with MH diameter ≤ 250 μm (0.48, 95% CI: 0.41–0.55; *I*^2^ = 0%; *P*_*het*_ = 0.62; Figure 5B) was higher than those with MH diameter of 250–400 μm (0.27, 95% CI: 0.21–0.34; *I*^2^ = 0%; *P*_*het*_ = 1.00; Figure 5C).

**Figure 6:**
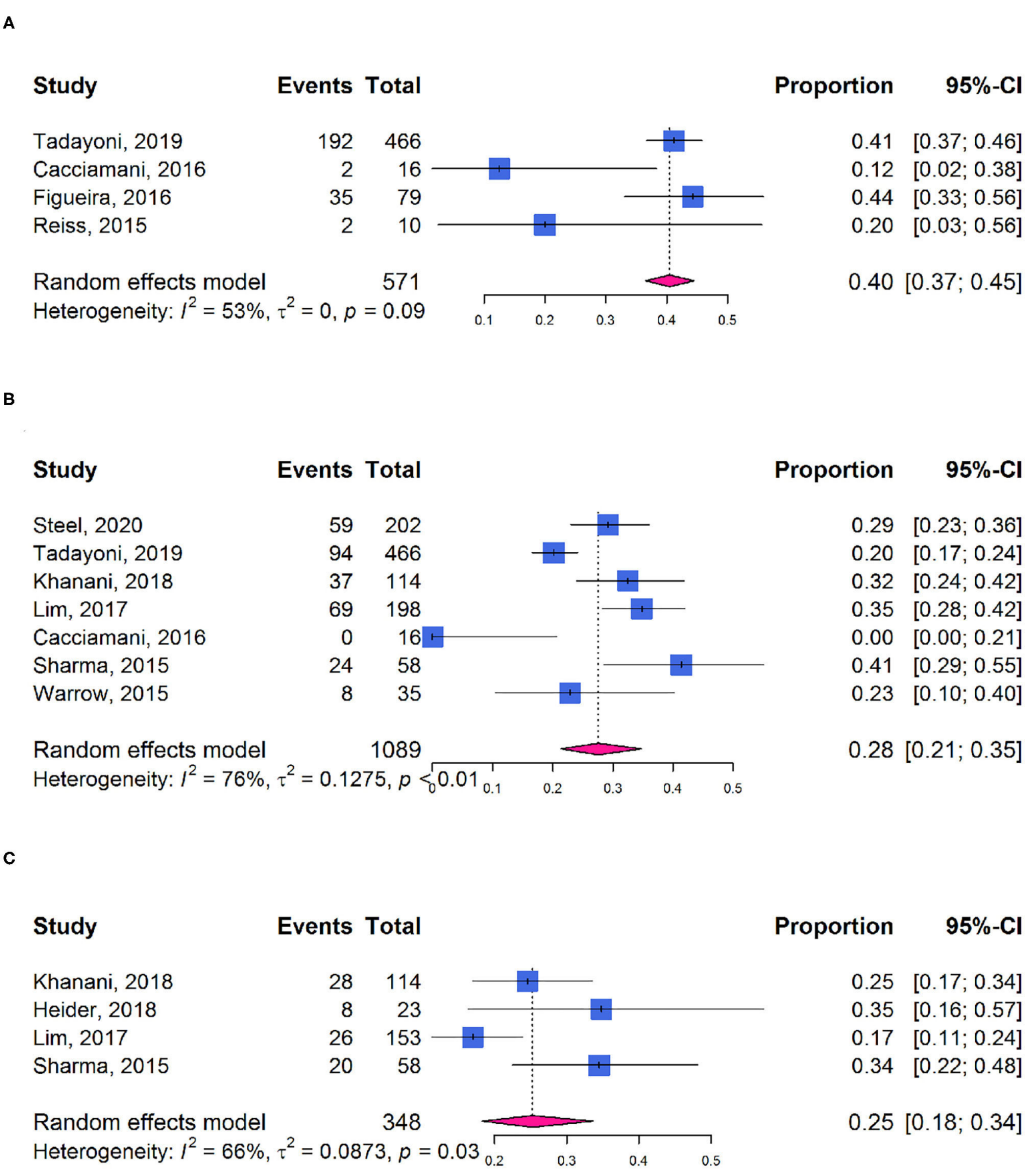
Forest plots of proportion of visual acuity improvement in participants receiving ocriplasmin therapy. **(A)** ≥1-line improvement in BCVA; **(B)** ≥2-line improvement in BCVA; **(C)** ≥3-line improvement in BCVA.

Approximately, 40% of participants showed at least 1-line improvement in BCVA after ocriplasmin treatment (95% CI: 0.37–0.45; *I*^2^ = 53%; *P*_*het*_ = 0.09; Figure 6A) and 28 or 25% of participants with at least 2-line (95% CI: 0.21–0.35; *I*^2^ = 76%; *P*_*het*_ < 0.01; Figure 6B) or 3-line (95% CI: 0.18–0.34; *I*^2^ = 66%; *P*_*het*_ = 0.03; Figure 6C) improvement in BCVA, respectively. Mean improvement was −0.13 logarithm of the minimum angle of resolution (logMAR) (95% CI: −0.17 to −0.08; *I*^2^ = 79%; *P*_*het*_ < 0.01; Supplementary Figure 3).

### Analysis of Potential Factors Affecting VMA Release and MH Closure

We further explored factors with potential to affect the rate of VMA release (VMAR) and MH closure including 556 participants from 14 studies. Participants who achieved VMAR were more likely to be female, without ERM, at a younger age, and with lower VMA diameter (Supplementary Table 2). Between participants with and without MH closure, MH closure was more likely to be achieved in participants with lower MH base diameter and minimum linear diameter (Supplementary Table 3).

In order to determine the optimal VMA profile of patient to receive ocriplasmin treatment, the ROC curve analysis was performed to find the predict ability of VMA diameter, age, ERM formation, and gender for VMA release in 5 studies including 120 participants (Supplementary Table 4). Cutoff values of VMA diameter and age were 506 μm (sensitivity: 81.13% and specificity: 56.41%) and 73 years (sensitivity: 66.18% and specificity: 57.69%; Supplementary Table 5), respectively. Specifically, the AUROC for VMA diameter < 506 μm, age < 73 years, without ERM formation, and female participants to predict VMAR were 0.71, 0.62, 0.62, and 0.59, respectively. These characteristics were entered into the multivariable logistic regression model resulted in an AUROC and its 95% CI being 0.84 (0.74–0.92). We listed the formula of *P*_*release*_ for calculating the estimated probability of VMAR using the character of patient including VMA diameter, sex, and ERM status. Also, we provided several examples and their *P*_*release*_ value for reference (Supplementary Table 6).

It has been recognized that sVMA may be associated with other conditions such as AMD, DR, or RVO. In addition to the above factors that directly affect the therapeutic effect, we also analyzed the causes of secondary VMA. Subgroup analysis was performed and stratified by the median proportions of AMD, DR, and RVO, all of which were found not to affect the rate of VMA release after ocriplasmin injection (Supplementary Table 7).

### Assessment of Study Quality and Publication Bias

Five RCTs were found to be of high quality. The OASIS trial (10) was found to have unclear risk of performance bias and the Novack et al. (18) study had unclear risk for selection, performance, and detection bias. The MIVI-IIT trial (9) showed unclear risk for selection and performance bias and the MIVI-TRUST trial (8) showed unclear risk for attrition bias (Supplementary Figure 4). In general, quality of the 50 cohort studies was found to be acceptable as shown in Supplementary Table 8. The included cohort studies showed relatively high quality in their objectives, statistical analyses, results, and conclusions (including follow-up and adverse events reporting), but performed less well on study design, intervention and cointervention, and outcome measures. No evidence of publication bias was found in analysis using the Begg's and Egger's tests and funnel plots (Supplementary Table 9; Supplementary Figures 5, 6).

## Discussion

We identified 5 RCTs and 50 cohort studies including 4,159 participants. Our results demonstrated that treatment with ocriplasmin increased the likelihood of VMA release and MH closure and was associated with improvements in BCVA and questionnaire-assessed visual function. No increased risk in overall AEs was found between ocriplasmin treatment and control. The results also showed that VMAR was more likely in patients with absence of ERM. Patients with smaller MH diameter were more likely to achieve MH closure. Our findings have comprehensively demonstrated the effectiveness of ocriplasmin in VMA and MH treatment as well as patient-related factors affecting outcomes and included guidance on selection of suitable patients to receive ocriplasmin treatment in clinic.

Posterior vitreous detachment is a physiological age-related process and incomplete PVD could cause VMA due to the persistent adhesion of the vitreous to the macula, especially the fovea. Persistent asymptomatic VMA may progress to VMT, also known as sVMA, causing retinal structure deformation such as macular edema and MH and accompanied by metamorphopsia, decreased visual acuity, and other symptoms. The RCTs included in this study indicated that treatment with ocriplasmin was more likely than control intervention to result in VMA release and MH closure, reduce the requirement for PPV, and achieve visual improvement, consistent with a previous meta-analysis (19). One RCT study on wAMD (18), showed that the VMA release of patients with wAMD was lower than previously demonstrated by other RCT studies, suggesting that VMA secondary to wAMD may be less responsive to ocriplasmin. However, this study found that ocriplasmin treatment and its causing VMA release decreased the number of antivascular endothelial growth factor injection in patients with wAMD.

Furthermore, we analyzed VFQ-25 composite scores changes found in the OASIS and the MIVI-TRUST trials (20, 21). Ocriplasmin treatment was associated with visual function improvement not only in BCVA, but also in this participant-reported questionnaire-based outcome. These scores reflect the influence of visual disability and visual symptoms on generic health domains and indicate the effect of treatment on activities related to daily visual functions (22, 23). The findings are relevant to clinical decision-making, since outcomes reported by patients are powerful tools to verify the effects of a treatment on health and daily-life activities of patients, both in terms of benefits and potential adverse effects.

The strengths of this meta-analysis include the comprehensive search strategy and retrieval of all the relevant trials and the focus on detecting the optimal patient profile for ocriplasmin treatment. In the UK, the National Institute for Health and Care Excellence (NICE) guidance recommends the use of ocriplasmin for treating VMT in adults without ERM, who have a MH ≤ 400 μm diameter and/or severe symptoms (24). Several studies have demonstrated that ocriplasmin therapy might be more beneficial for patients with sVMA with specific characteristics such as relatively small adhesion diameter and absence of ERM (25). More recently, Jackson et al. included 5 RCTs in an IPD meta-analysis and found that VMA release is more likely in younger, female patients and eyes with MH and less likely in the presence of ERM, broad VMA (>1,500 μm), DR, or pseudophakia (15). In this study, we also reported that patients with absence of ERM, the treatment of ocriplasmin was more likely to induce VMA release (Figure 4). Patients with MH were more likely to experience VMA release after ocriplasmin injection, since ERM and large VMA adhesion diameters seemed to be rare in the presence of MH. However, even if a patient with MH achieves VMA release, without MH closure, PPV is subsequently required to close the MH, which would still be considered a treatment failure. For patients with MH, small diameter MH (≤ 250 μm) was more likely to get nonsurgical closure (Figure 5). Therefore, as recommended by the NICE guidelines, patients with smaller MH may have a higher closure rate.

In this study, we extracted raw data from the included studies providing baseline characteristics of each participant and applied the ROC curves and the AUROCs analysis. By using the IPD of included studies, we estimated the cutoff values and evaluated the performance of these factors as predictors of VMA release after ocriplasmin therapy in a total of 120 patients. Further, the predict ability for female patients with VMA diameter < 506 μm and without ERM was 0.84. So, in clinical practice, when we encounter patients with sVMA and consider whether to use ocriplasmin for them, the gender of patient, ERM formation, and VMA diameter were brought into the formula (*P*_*Release*_). *P*_*Release*_ represents the estimated probability of VMA release. If *P*_*Release*_ is more than 0.68, the patient might probably achieve VMAR after with ocriplasmin therapy. We further provided several examples and their *P*_*release*_ value in Supplementary Table 6 for reference. These findings would help doctors about patient selection strategy.

It has been recognized that sVMA may be associated with other conditions such as AMD, DR, or RVO (18, 26, 27). These pathogenic factors may lead to an abnormally strong adhesion between the posterior vitreous cortex and macula. As mentioned earlier, the therapeutic effect of ocriplasmin for secondary VMA may be poorer than that for idiopathic VMA. In this study, we attempted to analyze whether AMD, DR, or RVO may affect the therapeutic effect of ocriplasmin, but a paucity of information on health status of patient in the included studies prevented this analysis.

Several other sVMA treatment modalities exist; observation often being the first approach. Studies report that 11 to 40% of sVMA cases resolve spontaneously (28, 29), with unpredictable timeframes. Moreover, sVMA may lead to anomalies of retinal morphology, being responsible for metamorphopsia or loss of visual acuity, which increases with duration. Previous meta-analyses evaluated VMA treatment by intravitreal gas injections and found VMAR in 84% and MH closure in 59% after perfluoropropane (C_3_F_8_) gas injection (30) or VMA resolution in 47% of cases with or without associated MH 1 month after the injection C_3_F8 or SF_6_ (31). Other studies have found VMA release in 36% of eyes treated with air injection (32). Recently, the first RCT for evaluating the safety and efficacy of intravitreal gas (C_3_F_8_) injection was terminated early because of safety concerns related to retinal detachments and retinal tears (33). So, the safety issue of intravitreal gas injection still requires great attention. More studies are needed to increase understanding of the benefits of different approaches to management of sVMA including observation, PPV, ocriplasmin, and intravitreal gas injections.

A potential limitation of this meta-analysis is that few trials compared different approaches of managing sVMA including PPV, intravitreal gas injection, ocriplasmin, and observation (32, 34, 35). It was, therefore, not possible for us to compare efficacy directly between these strategies. There might introduce some bias in the predict ability in IPD analysis for only based on 5 studies with limited sample size. Also, few studies observed recurrence after ocriplasmin therapy (36). In future studies, attention should be paid to recurrence rates and timeframes in ocriplasmin-induced patients with VMAR. In addition, highly myopic patients with VMA require special attention, since treatment may be challenging in this group (37). Insufficient study to date involves observation and follow-up after ocriplasmin treatment in this group of patients. Most of the existing study has been carried out in Europe and North America and the effects in other regions and races remain unclear. More long-term follow-up data and further analyses are needed to understand therapeutic effects in VMA induced by various etiologies.

## Conclusion

Evidence from the 5 RCTs and 50 cohort studies included here suggests that ocriplasmin is a suitable approach for treating sVMA. As clinicians, we should be increasingly cognizant of appropriate patient selection for ocriplasmin treatment and should take into account various factors such as MH, ERM, VMA diameter, age, and sex.

## Data Availability Statement

The original contributions presented in the study are included in the article/Supplementary Material, further inquiries can be directed to the corresponding author/s.

## Author Contributions

XC contributed to the study design, manuscript writing, literature search, data abstraction, and finalization. ML contributed to the literature review, data abstraction, manuscript writing, and statistical analysis. RY contributed to the literature review and statistical analysis. WW contributed to the manuscript writing and review. YW contributed to the study design, manuscript writing, and review. All authors contributed to the article and approved the submitted version.

## Funding

This study was supported by the National Natural Science Foundation of China under (Grant Nos. 82101128 and 81870686) and the Beijing Municipal Natural Science Foundation under (Grant No. 7184201). The funders had no role in the design or conduct of the study, collection, management, analysis, or interpretation of the data, preparation, review, or approval of the manuscript, or the decision to submit the manuscript for publication.

## Conflict of Interest

The authors declare that the research was conducted in the absence of any commercial or financial relationships that could be construed as a potential conflict of interest. The Reviewer FL declared shared affiliation with the authors to the handling Editor at time of review.

## Publisher's Note

All claims expressed in this article are solely those of the authors and do not necessarily represent those of their affiliated organizations, or those of the publisher, the editors and the reviewers. Any product that may be evaluated in this article, or claim that may be made by its manufacturer, is not guaranteed or endorsed by the publisher.
