# Supplementary material for: Efficacy and Safety of Ocriplasmin Use for Vitreomacular Adhesion and Its Predictive Factors: A Systematic Review and Meta-Analysis
Source: Front Med (Lausanne). 2022 Jan 13;8:759311. doi: 10.3389/fmed.2021.759311 (PMC8793778; doi:10.3389/fmed.2021.759311)
Supplement: Supplementary file 1 [file Data_Sheet_1.docx]

Supplementary Material

Efficacy and Safety of Ocriplasmin Use for Vitreomacular Adhesion and its Predictive Factors: a systematic review and meta-analysis

***Xi Chen, ^1^*^†^ Min Li, ^2†^ Ran You, ^1^ Wei Wang, ^1^ Yanling Wang, ^1^****

^1^Department of Ophthalmology, Beijing Friendship Hospital, Capital Medical University, Beijing, China

^2^Clinical Epidemiology and Evidence-Based Medicine Unit, National Clinical Research Center for Digestive Disease, Beijing Friendship Hospital, Capital Medical University, Beijing, China

***^†^*** These authors have contributed equally to this work

*** Correspondence:**

Xi Chen

xichen@ccmu.edu.cn

Yanling Wang
wangyanling999@vip.sina.com

***Supplementary Online Content List***

**Supplementary Figure 1.** Forest plots of risk for ocriplasmin injection in randomized control trails

**Supplementary Figure 2.** Forest plots of proportion of vitreomacular adhesion (VMA) release in participants with or without epiretinal membrane (ERM) at different time points after receiving ocriplasmin therapy

**Supplementary Figure 3.** Forest plots of mean change of logMAR in best-corrected visual acuity after ocriplasmin therapy in included cohort studies

**Supplementary Figure 4.** Quality assessment of included randomized control trails

**Supplementary Figure 5.** Funnel plots for publication bias assessment of included randomized control trails

**Supplementary Figure 6.** Funnel plots for publication bias assessment of included cohort studies

**Supplementary Table 1.** Characteristics of the 55 studies included in the meta-analysis

**Supplementary Table 2.** Characteristics of participants achieving or not achieving vitreomacular adhesion release after ocriplasmin treatment

**Supplementary Table 3.** Characteristics of participants achieving or not achieving macular hole (MH) closure after ocriplasmin treatment

**Supplementary Table 4.** Characteristics of the individual participant data analysis studies

**Supplementary Table 5.** Receiver operating characteristic curve analysis for predicting factors affecting vitreomacular adhesion release

**Supplementary Table 6.** Some examples in the predict model for vitreomacular adhesion release

**Supplementary Table 7.** Analysis of different etiologies affecting vitreomacular adhesion release

**Supplementary Table 8.** Quality assessment of included cohort studies

**Supplementary Table 9.** Publication bias assessment

**Supplementary File 1.** Searching strategy and result

**Supplementary File 2.** References to studies included in this review

**
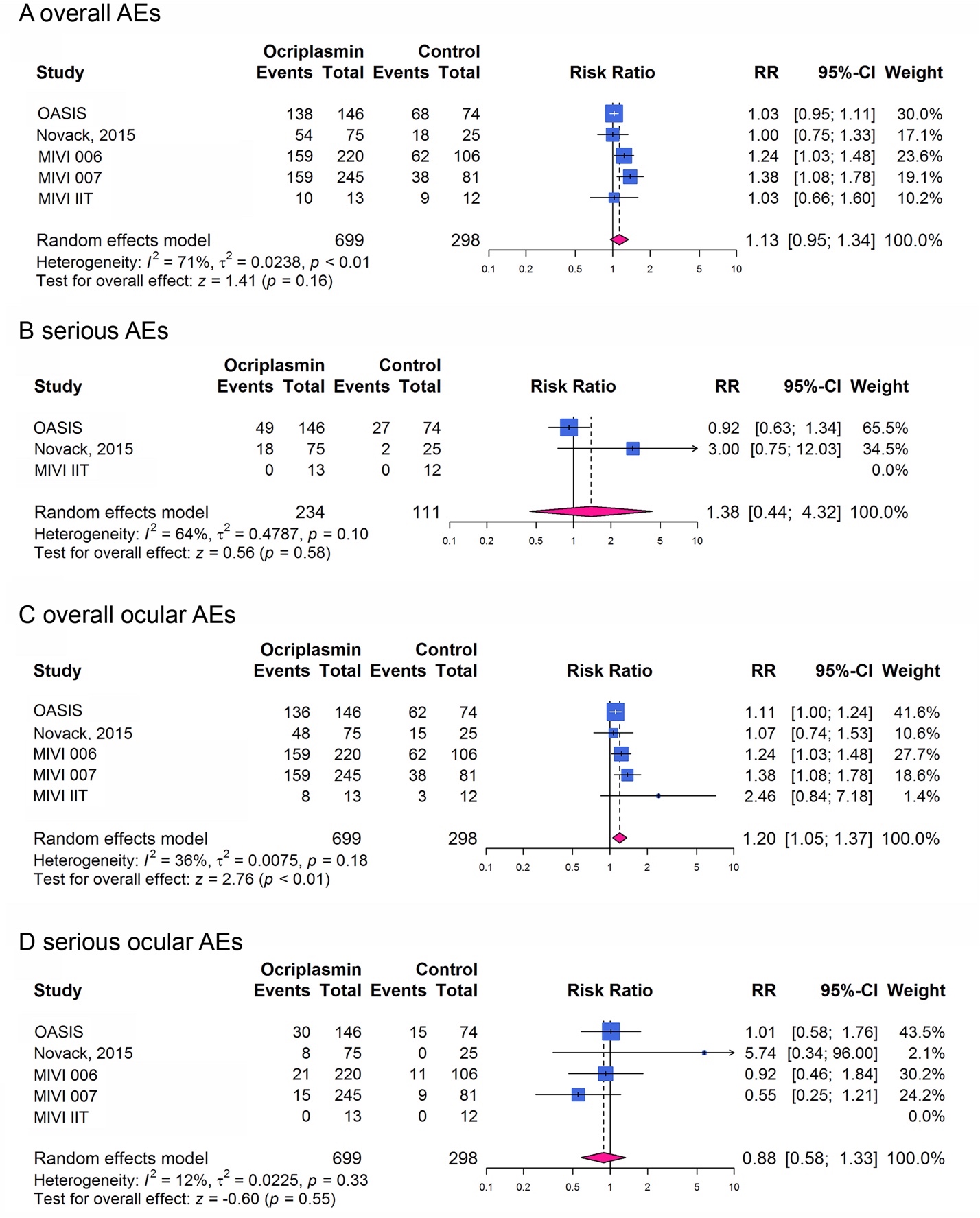
Supplementary Figure 1.** Forest plots of risk for ocriplasmin injection. A, comparison of all adverse events (AEs); B, comparison of serious AEs; C, comparison of all ocular AEs; D, comparison of serious ocular AEs.

**
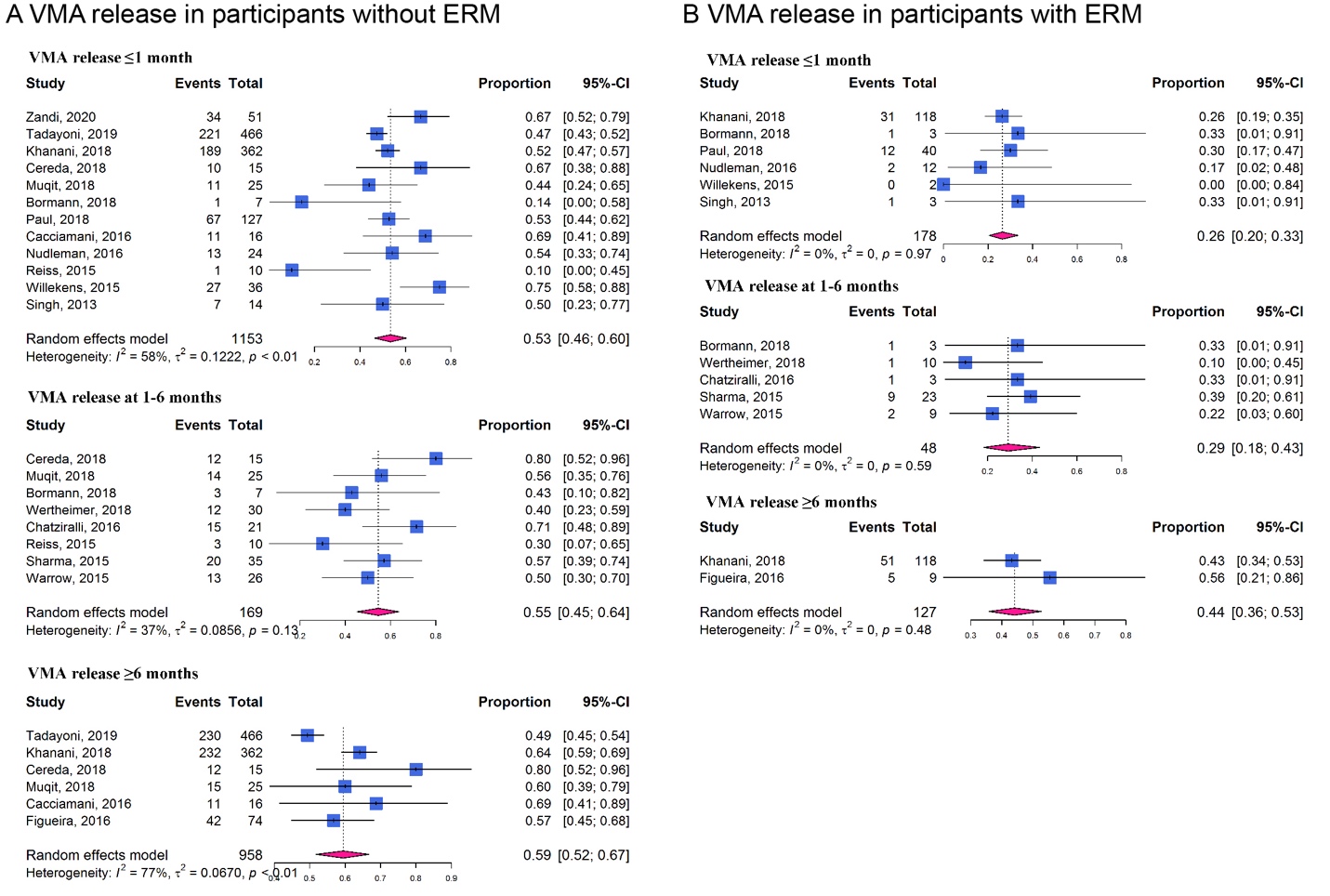
Supplementary Figure 2.** Forest plots of proportion of vitreomacular adhesion (VMA) release in participants with or without epiretinal membrane (ERM) at different time points after receiving ocriplasmin therapy. (**A**) in participants without ERM; (**B**) in participants with ERM

**
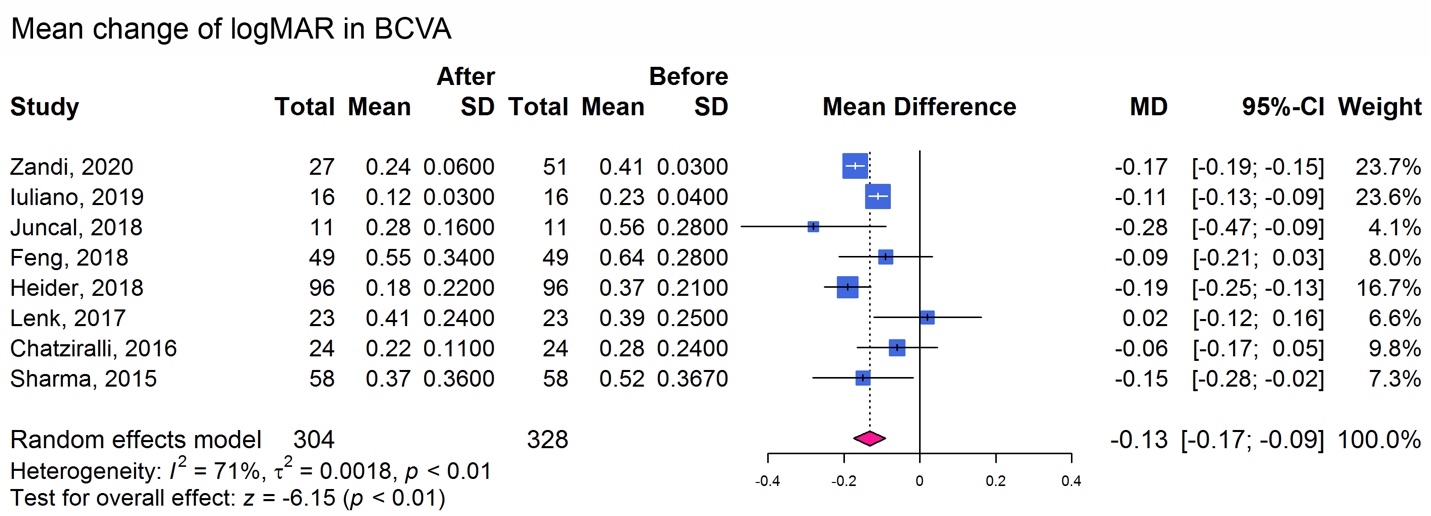
Supplementary Figure 3.** Forest plots of mean change of logMAR in best-corrected visual acuity after ocriplasmin therapy in included cohort studies


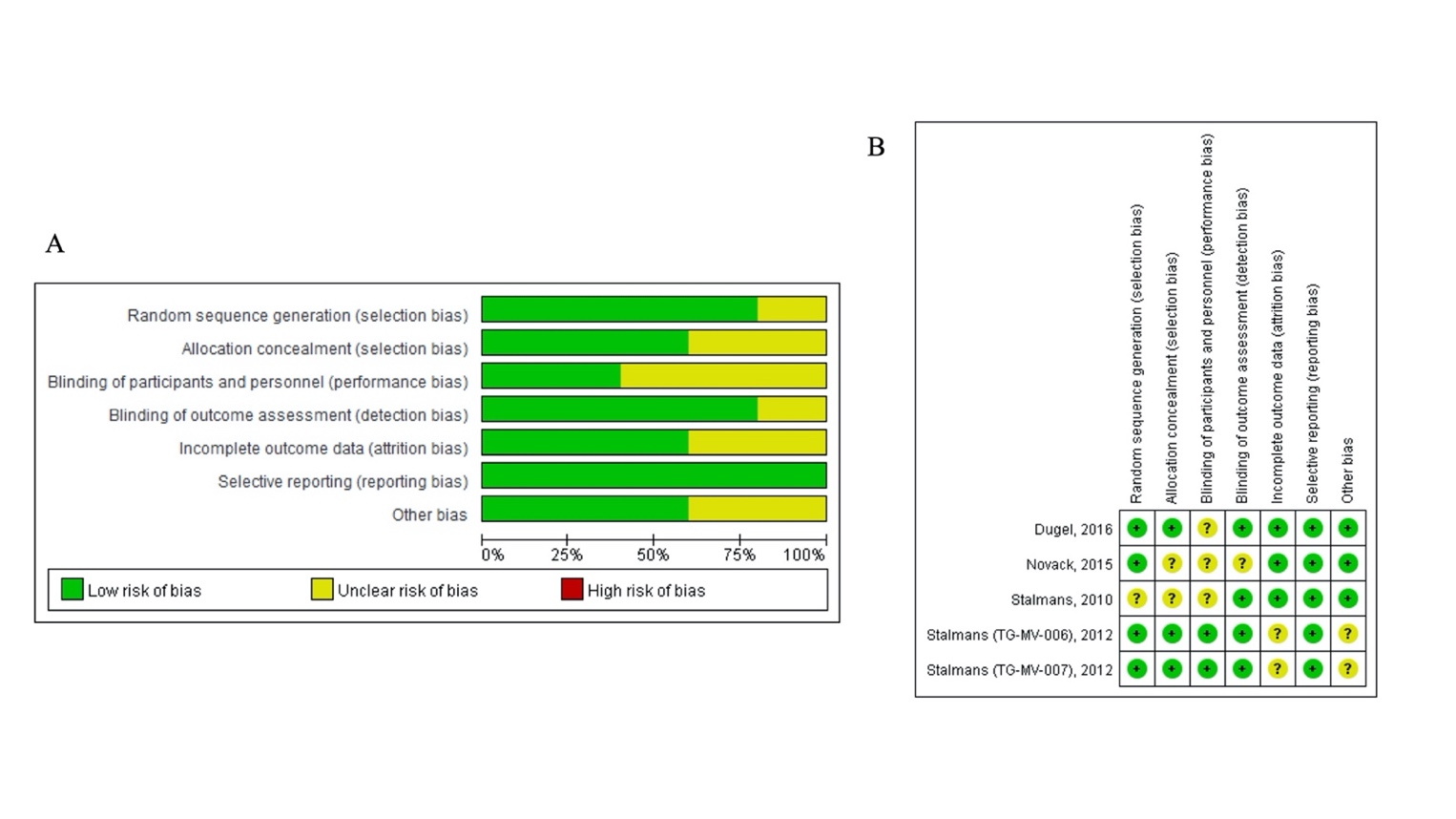


**Supplementary Figure 4. Quality assessment of included randomized control trails.** A, risk of bias item presented as percentages across included studies, B, list of each risk of bias item for each included study

**
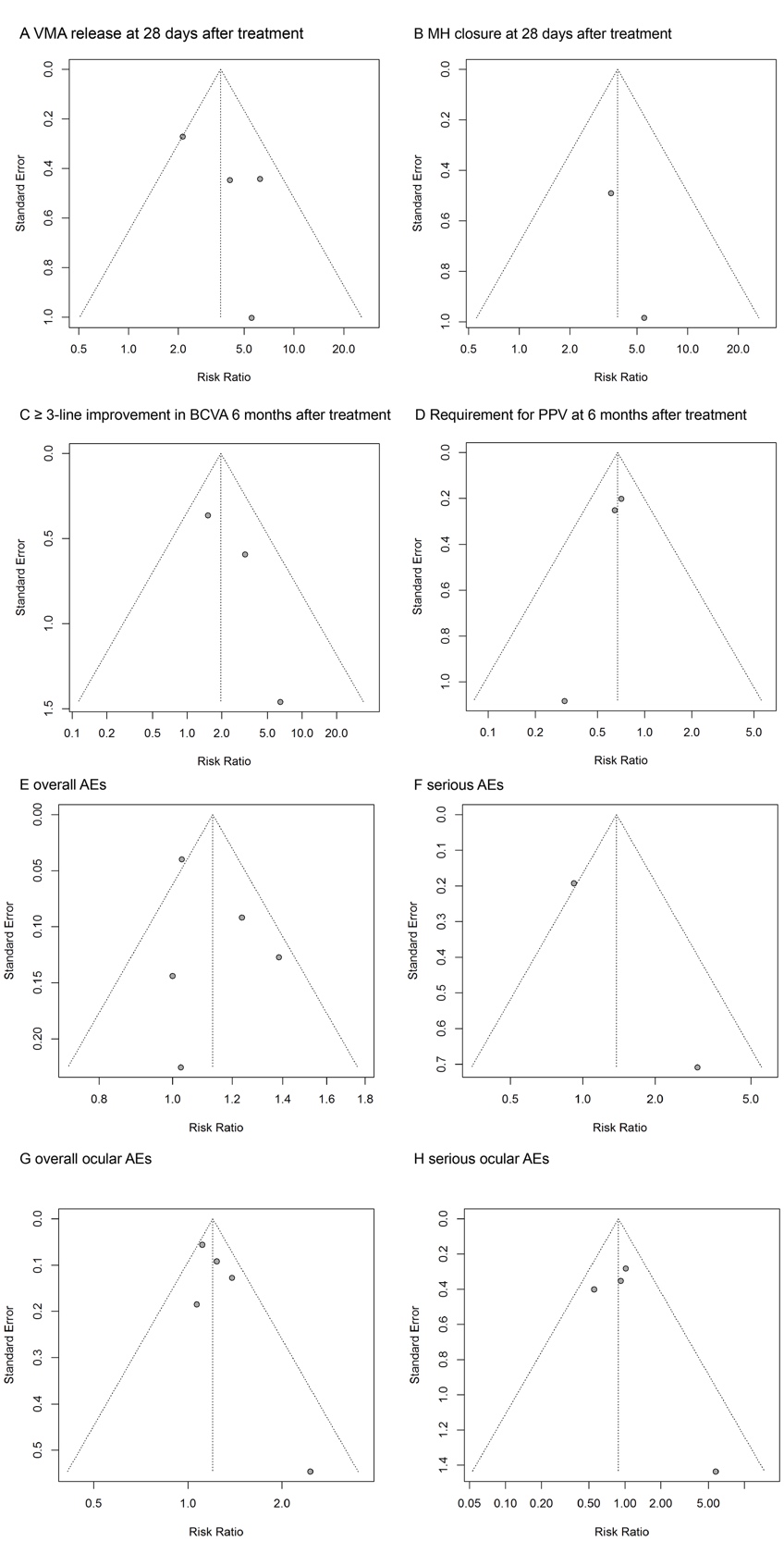
Supplementary Figure 5. Funnel plots for publication bias assessment of included randomized control trails.** A, for VMA release at 28 days after treatment; B, for macular hole (MH) closure at 28 days after treatment; C, for at least 3-line improvement in best-corrected visual acuity (BCVA) at 6 months after treatment; D, for incidence of pars plana vitrectomy (PPV) at 6 months after treatment; E, for incidence of all AEs; F, for incidence of any serious AEs; G, for incidence of all ocular AEs; H, for incidence of any serious ocular AEs.

**
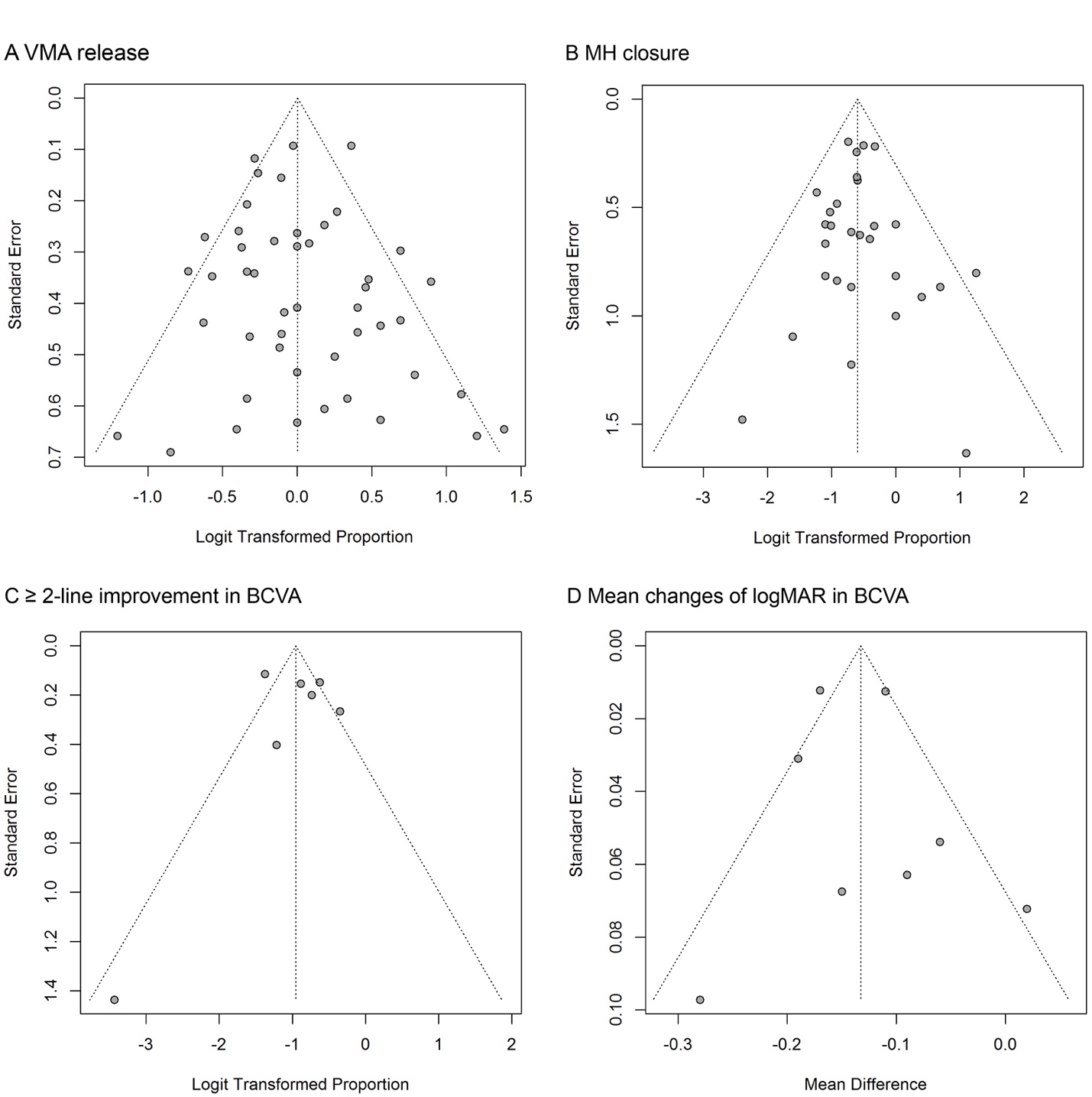
Supplementary Figure 6. Funnel plots for publication bias assessment of included cohort studies.** A, for proportion of VMA release; B, for proportion of MH closure; C, for proportion of ≥ 2-line improvement in BCVA; D, for mean changes of logMAR in BCVA.

**Supplementary Table 1. Characteristics of the 55 studies included in the meta-analysis**

| No | First author | Year | Country | Design | Sample size | Male/ Female | Age, year | Intervention | Follow-up duration |
| --- | --- | --- | --- | --- | --- | --- | --- | --- | --- |
| 1 | Dugel (1, 2) (OASIS) | 2016 | United States | RCT | 220 | 71/149 | 69.1 ± 10.34 | 125 μg ocriplasmin intravitreal injection or sham injection | 1 to 24 months |
| 2 | Novack (3) | 2015 | United States and Europe | RCT | 100 | 45/55 | 74.6 ± 7.86 | 125 μg ocriplasmin intravitreal injection or sham injection | 7 days to 12 months |
| 3 | Stalmans (4-6)  (MIVI 006) | 2012 | United States | RCT | 326 | 119/207 | 71.4 (range 18–96) | 125 μg ocriplasmin intravitreal injection or sham injection | 180 days |
| 4 | Stalmans (4-6)  (MIVI 007) | 2012 | United States and Europe | RCT | 326 | 104/222 | 72.0 (range 23–97) | 125 μg ocriplasmin intravitreal injection or sham injection | 180 days |
| 5 | Stalmans (7) (MIVI-IIT) | 2010 | Belgium | RCT | 25 | 11/14 | 72.1 (range 53–84) | Only include patients received 125 μg ocriplasmin intravitreal injection or sham injection | 3, 7, 14, 28, 90 and 180 days |
| 6 | Steel (8) (INJECT) | 2020 | Europe | Prospective cohort | 428 | 143/285 | 74.0 (quartile 67.0–79.0) | 125 μg ocriplasmin intravitreal injection | 14 and 28 days, 6, and 12 months |
| 7 | Zandi (9) | 2020 | United States | Retrospective case series | 51 | 19/32 | 76 ± 8 | 125 μg ocriplasmin intravitreal injection | 24 ± 14 months |
| 8 | Cacciamani (10) | 2020 | Italy | Prospective cohort | 23 | 5/18 | 69.5 ± 8.2 | 125 μg ocriplasmin intravitreal injection | 1 and 4 weeks |
| 9 | Tadayoni (11)(OVIID-1) | 2019 | Europe and Canada | Prospective cohort | 466 | 122/344 | 71.7 ± 8.3 | 125 μg ocriplasmin intravitreal injection | 28 to 180 days |
| 10 | Iuliano (12) | 2019 | Italy | Prospective cohort | 16 | 7/9 | 59.2 ± 3.4 | 125 μg ocriplasmin intravitreal injection | 1 month |
| 11 | Grinton ^a^ (13) | 2019 | England | Retrospective case series | 81 | NR | NR | ocriplasmin intravitreal injection | 4 weeks |
| 12 | Khanani (14) (ORBIT) | 2018 | United States | Prospective cohort | 480 | 171/309 | 71.3 ± 8.21 | 125 μg ocriplasmin intravitreal injection | 1 to 12 months |
| 13 | Cereda (15) | 2018 | Italy | Retrospective case series | 15 | 5/15 | 70 (range 45–84) | 125 μg ocriplasmin intravitreal injection | 1 week, 1, 3 and 6 months |
| 14 | Muqit (16)  (IPD study) | 2018 | United Kingdom | Retrospective case series | 25 | 8/17 | 71 | 125 μg ocriplasmin intravitreal injection | 4, 12, and 24 weeks |
| 15 | Bormann (17)  (IPD study) | 2018 | Germany | Retrospective case series | 10 | 3/7 | 75.1 (range 63–84) | 125 μg ocriplasmin intravitreal injection | 8 weeks |
| 16 | Feng (18) | 2018 | United States | Retrospective case series | 49 | 16/33 | 71 ± 10 | 125 μg ocriplasmin intravitreal injection | 10.3 ± 2.0 months |
| 17 | Paul (19) | 2018 | Germany | Retrospective case series | 167 | 49/118 | 72.7 ± 8.9 | ocriplasmin intravitreal injection | 28 ± 5 days |
| 18 | Wertheimer (20) | 2018 | Germany | Retrospective case series | 40 | 14/26 | 73 ± 9.5 | 125 μg ocriplasmin intravitreal injection | 1, 4, 12, and 24 weeks |
| 19 | Heider (21) | 2018 | Germany | Retrospective case series | 96 | 41/55 | 70.8 | ocriplasmin intravitreal injection | 7.13 ± 6.40 months |
| 20 | Wan ^a^ (22) | 2018 | Australia | Retrospective case series | 13 | NR | NR | ocriplasmin intravitreal injection | 7, 28, 90 and 180 days |
| 21 | Juncal (23) | 2018 | Canada | Retrospective case series | 11 | NR | 68.3 ± 8.74 | 125 μg ocriplasmin intravitreal injection | 13.6 (range 6-36) months |
| 22 | Manousaridis (24) | 2017 | Austria | Retrospective case series | 20 | NR | 68.5 (range 21–88) | 125 μg ocriplasmin intravitreal injection | 28 days |
| 23 | Lim (25) | 2017 | United States and United Kingdom | Retrospective case series | 208 | NR | NR | ocriplasmin intravitreal injection | at least 3 weeks |
| 24 | Scholz (26) | 2017 | Switzerland | Retrospective case series | 14 | 2/12 | 73 ± 10 | ocriplasmin intravitreal injection | 2 weeks and 4 months |
| 25 | Gkizis ^a^ (27) | 2017 | NR | Retrospective case series | 10 | 2/8 | NR | ocriplasmin intravitreal injection | 1, 7 and 28 days |
| 26 | Tacea ^a^ (28) | 2017 | United Kingdom | Retrospective case series | 33 | 10/23 | NR | ocriplasmin intravitreal injection | 1, 3 and 12 months |
| 27 | Zakri ^a^ (29) | 2017 | United Kingdom | Retrospective case series | 60 | 30/30 | 75 | ocriplasmin intravitreal injection | NR |
| 28 | Robson ^a^ (30) | 2017 | United Kingdom | Retrospective case series | 48 | 25/23 | 76.8 | ocriplasmin intravitreal injection | mean 39.8 days |
| 29 | Patel ^a^ (31) | 2017 | United States | Retrospective case series | 66 | NR | NR | 125 μg ocriplasmin intravitreal injection | 1 week, 1, 3, 6 and 12 months |
| 30 | Lenk (32) | 2017 | Germany | Retrospective case series | 21 | NR | NR | ocriplasmin intravitreal injection | NR |
| 31 | Itoh (33) | 2017 | United Kingdom | Retrospective case series | 19 | NR | NR | 125 μg ocriplasmin intravitreal injection | 1 week, 1 and 3 months |
| 32 | Cacciamani (34) | 2016 | Italy | Retrospective case series | 15 | 6/9 | 55.1 ± 4.6 | 125 μg ocriplasmin intravitreal injection | 6 months |
| 33 | Michalska-Malecka (35) | 2016 | Poland | Retrospective case series | 16 | NR | 75.6 ± 6.04 | 125 μg ocriplasmin intravitreal injection | 1 day, 1 week and 1 month |
| 34 | Tschuppert (36) | 2016 | Switzerland | Retrospective case series | 12 | NR | NR | 125 μg ocriplasmin intravitreal injection | 3, 10, 29 days to 4 months |
| 35 | Nudleman (37) | 2016 | United States | Retrospective case series | 35 | 10/25 | 74.1 (range 48.7–88.9) | ocriplasmin intravitreal injection | 1, 6 and 12 months |
| 36 | Steel (38) | 2016 | United Kingdom | Prospective cohort | 31 | 9/22 | 71 ± 6 | 125 μg ocriplasmin intravitreal injection | 4–6 weeks |
| 37 | Figueira (39) | 2016 | Portugal | Retrospective case series | 78 | 24/54 | 70.9 ± 8.7 | 125 μg ocriplasmin intravitreal injection | at least 1 month |
| 38 | Chatziralli (40) | 2016 | Greece | Prospective cohort | 24 | 8/16 | 71.4 ± 8.7 | 125 μg ocriplasmin intravitreal injection | 1, 7, 28 to 127 days |
| 39 | Sharma ^a^ (41) | 2016 | United States | Retrospective case series | 34 | NR | NR | ocriplasmin intravitreal injection | mean 13.6 months |
| 40 | Preziosa ^a^ (42) | 2016 | Italy | Retrospective case series | 13 | NR | NR | ocriplasmin intravitreal injection | 1 week, 1, 3 and 6 months |
| 41 | Atkins ^a^ (43) | 2016 | United Kingdom | Retrospective case series | 10 | NR | NR | 125 μg ocriplasmin intravitreal injection | 1 month |
| 42 | Steel (44)  (IPD study) | 2015 | United Kingdom | Prospective cohort | 12 | 3/9 | 72.6 ± 3.8 | 125 μg ocriplasmin intravitreal injection | 1 and 4 weeks |
| 43 | Quezada-Ruiz (45) | 2015 | United States | Retrospective case series | 23 | 6/17 | 74 (range 53–93) | ocriplasmin intravitreal injection | 174 (range 20–291) days |
| 44 | Reiss (46) | 2015 | United States | Retrospective case series | 10 | 4/6 | 73 ± 11.1 | ocriplasmin intravitreal injection | at least 28 days |
| 45 | Willekens (47)  (IPD study) | 2015 | Portugal | Retrospective case series | 37 | 8/29 | 73.7 ± 4.0 | ocriplasmin intravitreal injection | at least 28 days |
| 46 | Sharma (48) | 2015 | United States | Retrospective case series | 58 | 20/38 | 72 (range 59–94) | 125 μg ocriplasmin intravitreal injection | mean 8.7 months |
| 47 | Meyer ^a^ (49) | 2015 | United States | Retrospective case series | 22 | NR | NR | ocriplasmin intravitreal injection | at least 1 week |
| 48 | Warrow (50)  (IPD study) | 2015 | United States | Retrospective case series | 35 | 12/23 | 69.4 | 125 μg ocriplasmin intravitreal injection | 96.3 (range 7–211) days |
| 49 | Dyer ^a^ (51) | 2015 | United States | Retrospective case series | 34 | 19/15 | NR | ocriplasmin intravitreal injection | 1, 3, 6 and 12 months |
| 50 | Zhao ^a^ (52) | 2015 | United States | Retrospective case series | 19 | NR | NR | 125 μg ocriplasmin intravitreal injection | 7 days to 1 year |
| 51 | Coskey ^a^ (53) | 2014 | United States | Retrospective case series | 52 | NR | NR | ocriplasmin intravitreal injection | up to 9 months |
| 52 | Steinle ^a^ (54) | 2014 | United States | Retrospective case series | 24 | NR | 73 (range 53–93) | ocriplasmin intravitreal injection | 168 (range 20-291) days |
| 53 | Roth ^a^ (55) | 2014 | United States | Retrospective case series | 62 | NR | NR | 125 μg ocriplasmin intravitreal injection | 1 week and 1 month |
| 54 | Singh (56) | 2014 | United States | Retrospective case series | 17 | 6/11 | 68.8 ± 9.03 | 125 μg ocriplasmin intravitreal injection | 28 days |
| 55 | Kim ^a^ (57) | 2013 | United States | Retrospective case series | 19 | NR | NR | ocriplasmin intravitreal injection | NR |

Data were presented by percent or median (range)

Abbreviations: NR, not reported

^a^ Conference abstracts

**Supplementary Table 2**. Characteristics of participants achieving or not achieving vitreomacular adhesion release after ocriplasmin treatment

| Events | Estimation of merged value | |
| --- | --- | --- |
|  | VMA release | VMA persistence |
| Female (%) | 149/203 | 120/195 |
| Absence of ERM (%) | 153/171 | 123/170 |
| Age (y) | 69.29 (67.58, 71.00) | 74.90 (73.82, 75.97) |
| VMA diameter | 384.10 (314.88, 453.32) | 572.17 (460.00, 684.33) |
| CRT | 365.63 (338.72, 392.55) | 369.22 (342.03, 396.41) |

Abbreviations: VMA, vitreomacular adhesion; ERM, epiretinal membrane; CRT, central retinal thickness; data presents as mean (95% confidence intervals) for age, VMA diameter and CRT.

**Supplementary Table 3**. Characteristics of participants achieving or not achieving macular hole closure after ocriplasmin treatment

| Events | Estimation of merged value | |
| --- | --- | --- |
|  | MH closure | MH persistence |
| Female (%) | 19/24 | 30/39 |
| Age (y) | 67.42 (63.45, 71.38) | 72.55 (69.73, 75.36) |
| VMA diameter | 290.84 (266.09, 315.60) | 240.92 (122.04, 359.81) |
| MH base diameter | 307.56 (250.73, 364.39) | 649.91 (485.42, 814.41) |
| MH minimum linear diameter | 235.87 (188.61, 283.14) | 271.11 (231.10, 311.13) |

Abbreviations: MH, macular hole; VMA, vitreomacular adhesion; data presents as mean (95% confidence intervals) for VMA diameter, MH base diameter and MH minimum linear diameter.

**Supplementary Table 4**. **Characteristics of the individual participant data analysis studies**

| No | Study | Sample size | Male/ Female | Age, year | VMA release incidence | Proportion of MH | Proportion of ERM | VMA diameter  (mean ± SD) | Follow-up duration |
| --- | --- | --- | --- | --- | --- | --- | --- | --- | --- |
| 1 | Muqit, 2018 (16) | 25 | 8/17 | 71 | 15/25 | 6/25 | 0/25 | 524.7 ± 202.5 | 4, 12, and 24 weeks |
| 2 | Bormann, 2018 (17) | 10 | 3/7 | 75.1 (range 63–84) | 4/10 | 5/10 | 3/10 | NR | 8 weeks |
| 3 | Steel, 2015 (44) | 12 | 3/9 | 72.6 ± 3.8 | 7/12 | 12/12 | NR | 210.8 | 1 and 4 weeks |
| 4 | Willekens, 2015 (47) | 37 | 8/29 | 73.7 ± 4.0 | 27/37 | 10/37 | 2/37 | 387.2 ± 424.7 | at least 28 days |
| 5 | Warrow, 2015 (50) | 35 | 12/23 | 69.4 | 15/35 | 6/35 | 9/35 | 571.1 ± 439.7 | 96.3 (range 7–211) days |

Abbreviations: VMA, vitreomacular adhesion; MH, macular hole; ERM, epiretinal membrane; SD, standard deviation; NR, not reported

**Supplementary Table 5. Receiver operating characteristic curve analysis for predicting factors affecting vitreomacular adhesion release**

| Variables | Sample size | AUROC (95% CI) | Sensitivity | Specificity | Cut-off value |
| --- | --- | --- | --- | --- | --- |
| VMA diameter | 92 | 0.71 (0.61-0.80) | 81.13 (68.0-90.6) | 56.41 (39.6-72.2) | 506 |
| Age | 120 | 0.62 (0.53-0.71) | 66.18 (53.7-77.2) | 57.69 (43.2-71.3) | 73 |
| ERM formation | 83 | 0.62 (0.50-0.72) | 93.48 (82.1-98.6) | 29.73 (15.9-47.0) | No |
| Female | 120 | 0.59 (0.50-0.68) | 77.94 (66.2-87.1) | 40.38 (27.0-54.9) | No |
| MH | 120 | 0.57 (0.47-0.66) | 38.24 (26.7-50.8) | 75.00 (61.1-86.0) | No |
| Combined | 73 | 0.84 (0.74-0.92) | 73.81 (58.0-86.1) | 83.87 (66.3-94.5) | 0.68 |

*P_release_*=1/[1+exp(0.004*VMA diameter-1.896*female(1=Yes, 0=No)+2.174*ERM(1=Yes, 0=No) )]

Abbreviations: ROC, receiver operating characteristic curves; VMA, vitreomacular adhesion; AUROC, areas under ROCs; ERM, epiretinal membrane; MH, macular hole

**Supplementary Table 6. Some examples in the predict model for vitreomacular adhesion release**

| Covariate | Subject 1 | Subject 2 | Subject 3 | Subject 4 | Subject 5 | Subject 6 |
| --- | --- | --- | --- | --- | --- | --- |
| Age | 65 | 65 | 65 | 65 | 75 | 75 |
| Sex | Female | Male | Female | Male | Female | Male |
| VMA diameter (μm) | 100 | 100 | 300 | 300 | 600 | 600 |
| ERM | No | No | No | No | No | No |
| *P_release_* | 0.82 | 0.40 | 0.68 | 0.23 | 0.38 | 0.08 |

Abbreviations: VMA, vitreomacular adhesion; ERM, epiretinal membrane

**Supplementary Table 7. Analysis of different etiologies affecting vitreomacular adhesion release**

| Subgroup Analysis | No. of Studies | Rate of VMA Release | *I^2^* (%) |
| --- | --- | --- | --- |
| % of AMD |  |  |  |
| > 20% | 4 | 0.48 (0.39-0.57) | 0.0% |
| < 20% | 5 | 0.47 (0.36-0.57) | 73.2% |
| % of DR |  |  |  |
| > 10% | 6 | 0.44 (0.39-0.49) | 0.0% |
| < 10% | 5 | 0.44 (0.39-0.49) | 0.0% |
| % of RVO |  |  |  |
| > 4% | 2 | 0.43 (0.32-0.54) | 0.0% |
| < 4% | 1 | 0.43 (0.37-0.49) | - |

Abbreviations: VMA, vitreomacular adhesion; AMD, age-related macular degeneration; DR, diabetic retinopathy; RVO, retinal vein occlusion

**Supplementary Table 8. Quality assessment of included cohort studies**

| Study | Q1 | Q2 | Q3 | Q4 | Q5 | Q6 | Q7 | Q8 | Q9 | Q10 | Q11 | Q12 | Q13 | Q14 | Q15 | Q16 | Q17 | Q18 | Q19 | Q20 |
| --- | --- | --- | --- | --- | --- | --- | --- | --- | --- | --- | --- | --- | --- | --- | --- | --- | --- | --- | --- | --- |
| Steel, 2020 | Y | Y | Y | U | N | N | U | N | N | Y | U | Y | Y | Y | Y | N | N | Y | Y | N |
| Zandi, 2020 | Y | N | U | U | Y | Y | Y | Y | N | Y | U | Y | Y | Y | Y | Y | Y | Y | Y | Y |
| Cacciamani, 2020 | Y | Y | Y | U | Y | Y | Y | Y | N | Y | U | Y | Y | Y | Y | Y | Y | Y | Y | Y |
| Tadayoni, 2019 | Y | Y | Y | U | Y | Y | Y | Y | N | Y | U | Y | Y | Y | Y | Y | Y | Y | Y | Y |
| Iuliano, 2019 | Y | Y | N | Y | Y | Y | Y | Y | N | Y | U | Y | Y | Y | Y | Y | Y | N | Y | Y |
| Grinton, 2019 | Y | N | Y | U | Y | N | U | N | N | Y | U | Y | N | U | Y | Y | Y | N | Y | N |
| Khanani, 2018 | Y | Y | Y | Y | Y | Y | Y | Y | N | Y | U | Y | Y | Y | Y | Y | Y | Y | Y | Y |
| Cereda, 2018 | Y | N | N | U | Y | Y | Y | Y | N | Y | U | Y | Y | Y | Y | Y | N | Y | Y | P |
| Muqit, 2018 | Y | N | N | Y | Y | Y | Y | Y | N | Y | U | Y | Y | Y | Y | Y | N | Y | Y | Y |
| Bormann, 2018 | Y | N | N | U | Y | N | Y | Y | N | Y | U | Y | Y | Y | Y | Y | N | N | Y | P |
| Feng, 2018 | Y | N | N | Y | Y | Y | Y | Y | N | Y | U | Y | Y | Y | Y | Y | Y | Y | Y | Y |
| Paul, 2018 | Y | N | Y | Y | Y | Y | Y | N | N | N | U | Y | Y | Y | U | N | Y | N | Y | P |
| Wertheimer, 2018 | Y | N | N | Y | Y | Y | Y | Y | N | Y | U | Y | Y | Y | U | Y | Y | Y | Y | P |
| Heider, 2018 | Y | U | U | U | N | N | Y | N | N | N | U | Y | N | U | Y | Y | Y | N | Y | N |
| Wan, 2018 | Y | U | U | U | N | N | Y | N | N | Y | U | Y | Y | U | Y | N | Y | N | Y | N |
| Juncal, 2018 | Y | N | Y | U | Y | Y | Y | Y | N | Y | U | Y | Y | Y | Y | Y | Y | Y | Y | N |
| Manousaridis, 2017 | Y | N | N | U | Y | N | Y | Y | N | Y | U | Y | Y | Y | Y | Y | Y | Y | Y | Y |
| Lim, 2017 | Y | N | U | U | Y | N | Y | N | N | N | U | Y | Y | Y | Y | N | Y | Y | Y | Y |
| Itoh, 2017 | Y | N | N | Y | P | Y | Y | Y | N | Y | U | Y | Y | Y | Y | Y | Y | Y | Y | Y |
| Scholz, 2017 | Y | N | Y | U | Y | N | Y | N | N | Y | U | Y | Y | Y | Y | Y | Y | N | Y | P |
| Gkizis, 2017 | Y | U | N | U | Y | N | Y | N | N | P | U | Y | U | U | Y | Y | N | Y | Y | N |
| Tacea, 2017 | Y | N | U | U | Y | N | Y | N | N | P | U | Y | U | U | Y | Y | N | Y | Y | N |
| Zakri, 2017 | Y | N | Y | U | Y | N | Y | N | N | P | U | Y | U | U | U | N | N | N | Y | N |
| Robson, 2017 | Y | N | U | U | Y | N | Y | N | N | Y | U | Y | Y | U | Y | Y | Y | Y | Y | N |
| Patel, 2017 | Y | N | U | U | N | N | Y | Y | N | Y | U | Y | Y | U | Y | Y | N | N | Y | N |
| Lenk, 2017 | Y | N | U | U | N | N | Y | N | N | Y | U | Y | Y | Y | U | Y | Y | N | Y | N |
| Cacciamani, 2016 | Y | U | Y | Y | Y | Y | Y | Y | N | Y | U | Y | Y | Y | Y | Y | Y | Y | Y | Y |
| Michalska-Malecka, 2016 | Y | N | Y | U | Y | N | Y | Y | N | Y | U | Y | Y | U | Y | Y | Y | Y | Y | N |
| Tschuppert, 2016 | Y | N | U | Y | N | N | Y | Y | N | N | U | Y | N | U | Y | Y | Y | N | Y | P |
| Nudleman, 2016 | Y | N | N | U | Y | Y | Y | N | N | Y | U | Y | Y | U | Y | Y | Y | N | Y | Y |
| Steel, 2016 | Y | Y | Y | U | Y | P | Y | N | N | N | U | Y | Y | Y | Y | Y | Y | N | Y | P |
| Figueira, 2016 | Y | N | Y | U | Y | Y | Y | Y | N | N | U | Y | Y | Y | Y | Y | Y | N | Y | Y |
| Chatziralli, 2016 | Y | Y | Y | U | Y | Y | Y | Y | N | Y | U | Y | Y | Y | Y | Y | Y | Y | Y | P |
| Sharma, 2016 | Y | N | N | U | N | N | Y | N | N | P | U | Y | Y | U | Y | Y | N | N | Y | N |
| Preziosa, 2016 | Y | N | U | U | N | N | Y | N | N | Y | U | Y | Y | Y | Y | Y | N | N | Y | N |
| Atkins, 2016 | Y | N | U | U | N | N | Y | Y | N | P | U | Y | N | U | Y | N | N | Y | Y | N |
| Steel, 2015 | Y | Y | U | Y | Y | P | Y | Y | N | Y | U | Y | Y | Y | Y | Y | Y | N | Y | Y |
| Quezada-Ruiz, 2015 | Y | N | N | Y | Y | Y | Y | N | N | P | U | Y | Y | Y | Y | Y | Y | N | Y | P |
| Reiss, 2015 | Y | N | Y | Y | Y | Y | Y | N | N | Y | U | Y | Y | Y | Y | Y | N | N | Y | N |
| Willekens, 2015 | Y | N | N | U | Y | Y | Y | N | N | Y | U | Y | Y | Y | Y | Y | Y | Y | Y | Y |
| Sharma, 2015 | Y | N | N | Y | Y | Y | Y | Y | N | Y | U | Y | Y | Y | Y | Y | Y | Y | Y | Y |
| Meyer, 2015 | Y | N | Y | U | N | N | Y | N | N | N | U | Y | Y | U | U | Y | N | Y | Y | N |
| Warrow, 2015 | Y | N | Y | Y | Y | Y | Y | Y | N | Y | U | Y | Y | Y | Y | Y | Y | Y | Y | Y |
| Dyer, 2015 | Y | N | U | Y | P | N | Y | N | N | P | U | Y | Y | U | Y | Y | Y | N | Y | N |
| Zhao, 2015 | Y | N | U | U | N | N | Y | Y | N | N | U | Y | Y | Y | U | Y | Y | Y | Y | N |
| Coskey, 2014 | Y | N | Y | N | N | N | Y | N | N | N | U | Y | Y | U | U | Y | N | Y | Y | N |
| Steinle, 2014 | Y | N | U | Y | Y | N | Y | N | N | P | U | Y | Y | U | Y | Y | N | Y | Y | N |
| Roth, 2014 | Y | N | U | U | N | N | Y | Y | N | N | U | Y | U | U | Y | N | N | N | Y | N |
| Singh, 2014 | Y | N | Y | U | Y | Y | Y | Y | N | Y | U | Y | Y | U | Y | Y | N | Y | Y | P |
| Kim, 2013 | Y | N | N | N | N | N | Y | N | N | N | U | Y | U | U | U | Y | N | N | Y | N |

Green color represents for rating of “yes (Y)”. Yellow color represents for rating of “partial (P)/unclear (N)”. Red color represents for rating of “no (N)”.

**Supplementary Table 9. Publication bias assessment**

|  | Egger’s Test | |  | Begg’ Test | |
| --- | --- | --- | --- | --- | --- |
|  | t | *p* |  | Z | *p* |
| RCTs |  |  |  |  |  |
| VMA release | 1.42 | 0.29 |  | -0.34 | 1.00 |
| MH closure | - | - |  | 0.00 | 1.00 |
| Vision improvement | 2.00 | 0.30 |  | 0.00 | 1.00 |
| Incidence of vitrectomy | -5.03 | 0.13 |  | 1.04 | 0.30 |
| Incidence of all AEs | 0.97 | 0.40 |  | 0.24 | 0.81 |
| Incidence of any serious AE | - | - |  | 0.00 | 1.00 |
| Incidence of all ocular AE | 1.68 | 0.19 |  | 0.73 | 0.46 |
| Incidence of any serious ocular AEs | 0.80 | 0.51 |  | -0.34 | 1.00 |
| Cohort studies |  |  |  |  |  |
| Proportion of VMA release | 0.25 | 0.81 |  | 1.23 | 0.22 |
| Proportion of MH closure | 1.09 | 0.29 |  | 1.82 | 0.07 |
| Proportion of ≥ 2-line improvement in BCVA | 1.99 | 0.12 |  | 0.38 | 0.71 |
| Mean changes of logMAR in BCVA | -0.37 | 0.72 |  | 0.12 | 0.90 |

Abbreviations: VMA, vitreomacular adhesion; MH, macular hole; AEs, adverse events; BCVA, best-corrected visual acuity

**Supplementary File 1. Searching strategy and result**

**Number of citations by each database searched**

| **Databases** | **Citations** |
| --- | --- |
| PubMed | 624 |
| EMBASE | 483 |
| Cochrane Library | 77 |
| Total databases | 1184 (with 235 duplicates) |
| Total databases after removing duplicates | 949 |

**Searching strategy for PubMed**

1. "vitreous body" [MeSH Terms]
2. "vitreous detachment" [MeSH Terms]
3. "retinal perforations" [MeSH Terms]
4. "tissue adhesions" [MeSH Terms]
5. vitreomacular adhesion [Title/Abstract]
6. vitreomacular traction [Title/Abstract]
7. macula hole [Title/Abstract]
8. VMA [Title/Abstract]
9. VMT [Title/Abstract]
10. MH [Title/Abstract]
11. 1 OR 2 OR 3 OR 4 OR 5 OR 6 OR 7 OR 8 OR 9 OR 10
12. "fibrinolysin" [MeSH Terms]
13. "fibrinolytic agents" [MeSH Terms]
14. "proteolysis" [MeSH Terms]
15. "peptide fragments" [MeSH Terms]
16. ocriplasmin [Title/Abstract]
17. Jetrea [Title/Abstract]
18. Microplasmin [Title/Abstract]
19. 12 OR 13 OR 14 OR 15 OR 16 OR 17 OR 18
20. 11 AND 19

**Search strategy for EMBASE**

1. 'vitreous body'/exp/mj
2. 'vitreous detachment'/exp/mj
3. 'retinal perforations'/exp/mj
4. 'tissue adhesions'/exp/mj
5. 'vitreomacular adhesion':ab,ti
6. 'vitreomacular traction':ab,ti
7. 'macula hole':ab,ti
8. 'VMA':ab,ti
9. 'VMT':ab,ti
10. 'MH':ab,ti
11. 1 OR 2 OR 3 OR 4 OR 5 OR 6 OR 7 OR 8 OR 9 OR 10
12. 'fibrinolysin'/exp/mj
13. 'fibrinolytic agents'/exp/mj
14. 'proteolysis'/exp/mj
15. 'peptide fragment'/exp/mj
16. 'ocriplasmin':ab,ti
17. 'Jetrea':ab,ti
18. 'Microplasmin':ab,ti
19. 12 OR 13 OR 14 OR 15 OR 16 OR 17 OR 18
20. 11 AND 19

**Search strategy for Cochrane Library**

1. Mesh Descriptor: [vitreous body] explode all trees
2. Mesh Descriptor: [vitreous detachment] explode all trees
3. Mesh Descriptor: [retinal perforations] explode all trees
4. Mesh Descriptor: [tissue adhesions] explode all trees
5. (vitreomacular adhesion) :ti,ab,kw
6. (vitreomacular traction) :ti,ab,kw
7. (macula hole) :ti,ab,kw
8. (VMA) :ti,ab,kw
9. (VMT) :ti,ab,kw
10. (MH) :ti,ab,kw
11. 1 OR 2 OR 3 OR 4 OR 5 OR 6 OR 7 OR 8 OR 9 OR 10
12. Mesh Descriptor: [fibrinolysin] explode all trees
13. Mesh Descriptor: [fibrinolytic agents] explode all trees
14. Mesh Descriptor: [proteolysis] explode all trees
15. Mesh Descriptor: [peptide fragment] explode all trees
16. (ocriplasmin) :ti,ab,kw
17. (Jetrea) :ti,ab,kw
18. (Microplasmin) :ti,ab,kw
19. 12 OR 13 OR 14 OR 15 OR 16 OR 17 OR 18
20. 11 AND 19

**Supplementary File 2. References to studies included in this review**

1. Dugel PU, Tolentino M, Feiner L, Kozma P, Leroy A. Results of the 2-Year Ocriplasmin for Treatment for Symptomatic Vitreomacular Adhesion Including Macular Hole (OASIS) Randomized Trial. *Ophthalmology*. (2016) 123:2232-47. doi: 10.1016/j.ophtha.2016.06.043

2. Mein C, Dugel PU, Feiner L, Drenser K, Miller D, Benz M, et al. Patient-reported visual function from the ocriplasmin for treatment for symptomatic vitreomacular adhesion, including macular hole (OASIS) study. *Retina (Philadelphia, Pa)*. (2020) 40:1331-8. doi: 10.1097/iae.0000000000002599

3. Novack RL, Staurenghi G, Girach A, Narendran N, Tolentino M. Safety of intravitreal ocriplasmin for focal vitreomacular adhesion in patients with exudative age-related macular degeneration. *Ophthalmology*. (2015) 122:796-802. doi: 10.1016/j.ophtha.2014.10.006

4. Stalmans P, Benz MS, Gandorfer A, Kampik A, Girach A, Pakola S, et al. Enzymatic vitreolysis with ocriplasmin for vitreomacular traction and macular holes. *The New England journal of medicine*. (2012) 367:606-15. doi: 10.1056/NEJMoa1110823

5. Varma R, Haller JA, Kaiser PK. Improvement in patient-reported visual function after ocriplasmin for vitreomacular adhesion: results of the microplasmin for intravitreous injection-traction release without surgical treatment (MIVI-TRUST) trials. *JAMA ophthalmology*. (2015) 133:997-1004. doi: 10.1001/jamaophthalmol.2015.1746

6. Haller JA, Stalmans P, Benz MS, Gandorfer A, Pakola SJ, Girach A, et al. Efficacy of intravitreal ocriplasmin for treatment of vitreomacular adhesion: subgroup analyses from two randomized trials. *Ophthalmology*. (2015) 122:117-22. doi: 10.1016/j.ophtha.2014.07.045

7. Stalmans P, Delaey C, de Smet MD, van Dijkman E, Pakola S. Intravitreal injection of microplasmin for treatment of vitreomacular adhesion: results of a prospective, randomized, sham-controlled phase II trial (the MIVI-IIT trial). *Retina (Philadelphia, Pa)*. (2010) 30:1122‐7. doi: 10.1097/IAE.0b013e3181e0970a

8. Steel DHW, Patton N, Stappler T, Karia N, Hoerauf H, Patel N, et al. Ocriplasmin for vitreomacular traction in clinical practice: the INJECT study. *Retina (Philadelphia, Pa)*. (2021) 41:266-76. doi: 10.1097/IAE.0000000000002862

9. Zandi S, Freiberg F, Vaclavik V, Pfister IB, Traine PG, Kaya C, et al. Morphological Reconstitution and Persistent Changes After Intravitreal Ocriplasmin for Vitreomacular Traction and Macular Hole. *Journal of ocular pharmacology and therapeutics.* (2020) 36:126-32. doi: 10.1089/jop.2019.0051

10. Cacciamani A, Gattegna R, Pileri M, Di Nicola M, Bardanzellu S, Facciolo G, et al. Short-term changes in posterior vitreous cortex following intravitreal ocriplasmin for symptomatic vitreomacular traction syndrome: a prospective study. *International ophthalmology*. (2020) 40:185-93. doi: 10.1007/s10792-019-01177-7

11. Tadayoni R, Holz FG, Zech C, Liu X, Spera C, Stalmans P. Assessment of anatomical and functional outcomes with ocriplasmin treatment in patients with vitreomacular traction with or without macular holes: Results of OVIID-1 Trial. *Retina (Philadelphia, Pa)*. (2019) 39:2341-52. doi: 10.1097/iae.0000000000002332

12. Iuliano L, Fogliato G, Colombo R, Sacconi R, Querques G, Bandello F, et al. Reduced perfusion density of superficial retinal capillary plexus after intravitreal ocriplasmin injection for idiopathic vitreomacular traction. *BMC ophthalmology*. (2019) 19:108. doi: 10.1186/s12886-019-1119-9

13. Grinton M, Rees J, Habib MS, Hillier R, Vaideanu-Collins D, Jonathan S, et al. Predictive factors for successful treatment with intravitreal ocriplasmin. *Investigative Ophthalmology and Visual Science*. (2019) 60.

14. Khanani AM, Duker JS, Heier JS, Kaiser PK, Joondeph BC, Kozma P, et al. Ocriplasmin Treatment Leads to Symptomatic Vitreomacular Adhesion/Vitreomacular Traction Resolution in the Real-World Setting: The Phase IV ORBIT Study. *Ophthalmology Retina*. (2019) 3:32-41. doi: 10.1016/j.oret.2018.07.011

15. Cereda MG, Preziosa C, DʼAgostino I, Cozzi M, Bottoni F, Pellegrini M, et al. Ocriplasmin for vitreomacular traction: looking outside the macula: a wide-field optical coherence tomography study. *Retina (Philadelphia, Pa)*. (2018) 38:1541-8. doi: 10.1097/iae.0000000000001785

16. Muqit MMK, Hamilton R, Ho J, Tucker S, Buck H. Intravitreal ocriplasmin for the treatment of vitreomacular traction and macular hole- A study of efficacy and safety based on NICE guidance. *PloS one*. (2018) 13:e0197072. doi: 10.1371/journal.pone.0197072

17. Bormann C, Apitzsch BC, Habermann A, Hammer U, Hammer T. Experience with ocriplasmin in patients with vitreomacular traction syndrome: a retrospective study of 10 patients. *Retinal cases & brief reports*. (2018). doi: 10.1097/icb.0000000000000717

18. Feng HL, Roth DB, Hasan A, Fine HF, Wheatley HM, Prenner JL, et al. Intravitreal ocriplasmin in clinical practice: predictors of success, visual outcomes, and complications. *Retina (Philadelphia, Pa)*. (2018) 38:128-36. doi: 10.1097/iae.0000000000001505

19. Paul C, Heun C, Müller HH, Hoerauf H, Feltgen N, Wachtlin J, et al. Calculating the individual probability of successful ocriplasmin treatment in eyes with VMT syndrome: a multivariable prediction model from the EXPORT study. *The British journal of ophthalmology*. (2018) 102:1092-7. doi: 10.1136/bjophthalmol-2017-310874

20. Wertheimer C, Haritoglou C, Laubichler P, Wolf A, Kaessmann K, Schumann RG, et al. Impact of Preinjection Spectral Domain Optical Coherence Tomography Findings in the Use of Intravitreal Ocriplasmin in a Clinical Setting. *International journal of ophthalmology Zeitschrift fur Augenheilkunde*. (2018) 239:11-8. doi: 10.1159/000480406

21. Heider A, Dimopoulos S, Szurman P, Januschowski K. Real-life experience with intravitreal ocriplasmin on vitreomacular traction and full-thickness macular holes. *Acta ophthalmologica*. (2018) 96:e890-e1. doi: 10.1111/aos.13199

22. Wan R, Hong T, Chang A. Structural and functionalassessment in vitreomaculartraction following ocriplasmintreatment. *Clinical and Experimental Ophthalmology*. (2018) 46:120-1. doi: 10.1111/ceo.13405

23. Juncal VR, Chow DR, Vilà N, Kapusta MA, Williams RG, Kherani A, et al. Ocriplasmin versus vitrectomy for the treatment of macular holes. *Canadian journal of ophthalmology*. (2018) 53:441-6. doi: 10.1016/j.jcjo.2018.01.017

24. Manousaridis K, Peter-Reichart S, Mennel S. Ocriplasmin treatment for vitreomacular traction in real life: can the indication spectrum be expanded? *Graefe's archive for clinical and experimental ophthalmology*. (2017) 255:1907-16. doi: 10.1007/s00417-017-3731-9

25. Lim JI, Glassman AR, Aiello LP, Chakravarthy U, Flaxel CJ, Singerman LJ, et al. Macula Society Collaborative Retrospective Study of Ocriplasmin for Symptomatic Vitreomacular Adhesion. *Ophthalmology Retina*. (2017) 1:413-20. doi: 10.1016/j.oret.2016.10.018

26. Scholz P, Sitnilska V, Hess J, Becker M, Michels S, Fauser S. Comparison of resolution of vitreomacular traction after ocriplasmin treatment or vitrectomy. *Retina (Philadelphia, Pa)*. (2019) 39:180-5. doi: 10.1097/iae.0000000000001926

27. Gkizis I, Garnavou-Xirou C, Velissaris S, Kabanarou S, Chatziralli I, Kontou E, et al. Enzymatic vitreolysis with ocriplasmin for symptomatic vitreomacular traction syndrome. *Acta ophthalmologica*. (2017) 95. doi: 10.1111/j.1755-3768.2017.0F057

28. Tacea F, Makris L, Kamal A. One year retrospective analysis of ocriplasmin for the treatment of symptomatic vitreomacular traction. *Investigative Ophthalmology and Visual Science*. (2017) 58.

29. Zakri RH, Lee H, Patel N. Analysis of foveal angle after successful VMT detachment. *Investigative Ophthalmology and Visual Science*. (2017) 58.

30. Robson C, Patel N. The 'halo' and 'inverted fovea'; Novel three-dimensional signs of vitreomacular traction on optical coherence topography. *Investigative Ophthalmology and Visual Science*. (2017) 58.

31. Patel D, Shah A, Melchioris A, Miller DG. Evaluation of visual acuity and acute retinal changes following intravitreal injection of ocriplasmin. *Investigative Ophthalmology and Visual Science*. (2017) 58.

32. Lenk J, Matthé E, Ventzke S, Pillunat LE, Sandner D. [Initial Clinical Experiences Using Ocriplasmin for the Treatment of Vitreomacular Traction with or without a Macular Hole]. *Klinische Monatsblatter fur Augenheilkunde*. (2018) 235:73-80. doi: 10.1055/s-0042-124511

33. Itoh Y, Ehlers JP. Ellipsoid zone mapping and outer retinal characterization after intravitreal ocriplasmin. *Retina (Philadelphia, Pa)*. (2016) 36:2290-6. doi: 10.1097/iae.0000000000001110

34. Cacciamani A, Gelso A, Simonett JM, Ripandelli G, Pileri M, Stirpe M, et al. Longitudinal microperimetry evaluation after intravitreal ocriplasmin injection for vitreomacular traction. *Retina (Philadelphia, Pa)*. (2017) 37:1832-8. doi: 10.1097/iae.0000000000001432

35. Michalska-Małecka K, Witek K, Sierocka-Stępień J, Wyględowska-Promieńska D, Nowak M. Novel possibility of vitreomacular traction treatment. *Acta ophthalmologica*. (2016) 94:e818-e9. doi: 10.1111/aos.13075

36. Tschuppert S, Gerding H. Transient subfoveal fluid and visual loss after ocriplasmin. *Klinische Monatsblatter fur Augenheilkunde*. (2016) 233:453-5. doi: 10.1055/s-0041-111807

37. Nudleman E, Franklin MS, Wolfe JD, Williams GA, Ruby AJ. Resolution of subretinal fluid and outer retinal changes in patients treated with ocriplasmin. *Retina (Philadelphia, Pa)*. (2016) 36:738-43. doi: 10.1097/iae.0000000000000755

38. Steel DH, Parkes C, Papastavrou VT, Avery PJ, El-Ghrably IA, Habib MS, et al. Predicting macular hole closure with ocriplasmin based on spectral domain optical coherence tomography. *Eye (London, England)*. (2016) 30:740-5. doi: 10.1038/eye.2016.42

39. Figueira J, Martins D, Pessoa B, Ferreira N, Meireles A, Sampaio A, et al. The Portuguese Experience with Ocriplasmin in Clinical Practice. *Ophthalmic research*. (2016) 56:186-92. doi: 10.1159/000446842

40. Chatziralli I, Theodossiadis G, Parikakis E, Datseris I, Theodossiadis P. Real-life experience after intravitreal ocriplasmin for vitreomacular traction and macular hole: a spectral-domain optical coherence tomography prospective study. *Graefe's archive for clinical and experimental ophthalmology*. (2016) 254:223-33. doi: 10.1007/s00417-015-3031-1

41. Sharma P, Rahimy E, Regillo CD. Pharmacologic closure rate of full thickness macular hole with ocriplasmin-1 year follow-up data. *Investigative Ophthalmology and Visual Science*. (2016) 57:4046.

42. Preziosa C, D'Agostino I, Nava U, Erba S, Cereda MG, Bottoni F, et al. Ocriplasmin for vitreo-macular traction: A Wide-Field OCT Study. *Investigative Ophthalmology and Visual Science*. (2016) 57:4047.

43. Atkins K, Taylor S. Clinical results of Ocriplasmin versus C3F8 gas for symptomatic Vitreomacular Traction Syndrome. *Investigative Ophthalmology and Visual Science*. (2016) 57:4049.

44. Steel DH, Sandinha MT, White K. The Plane of Vitreoretinal Separation and Results of Vitrectomy Surgery in Patients Given Ocriplasmin for Idiopathic Macular Hole. *Investigative ophthalmology & visual science*. (2015) 56:4038-44. doi: 10.1167/iovs.15-16409

45. Quezada-Ruiz C, Pieramici DJ, Nasir M, Rabena M, Steinle N, Castellarin AA, et al. Outer retina reflectivity changes on sd-oct after intravitreal ocriplasmin for vitreomacular traction and macular hole. *Retina (Philadelphia, Pa)*. (2015) 35:1144-50. doi: 10.1097/iae.0000000000000544

46. Reiss B, Smithen L, Mansour S. Transient vision loss after ocriplasmin injection. *Retina (Philadelphia, Pa)*. (2015) 35:1107-10. doi: 10.1097/iae.0000000000000542

47. Willekens K, Abegão Pinto L, Vandewalle E, Stalmans I, Stalmans P. Improved efficacy of ocriplasmin for vitreomacular traction release and transient changes in optic disk morphology. *Retina (Philadelphia, Pa)*. (2015) 35:1135-43. doi: 10.1097/iae.0000000000000507

48. Sharma P, Juhn A, Houston SK, Fineman M, Chiang A, Ho A, et al. Efficacy of intravitreal ocriplasmin on vitreomacular traction and full-thickness macular holes. *American journal of ophthalmology*. (2015) 159:861-7.e2. doi: 10.1016/j.ajo.2015.01.034

49. Meyer JC, Shah GK, Blinder KJ, Waheed NK, Reichel E, Stalmans P, et al. Early evolution of the vitreomacular interface and clinical efficacy after ocriplasmin injection for symptomatic vitreomacular adhesion. *Ophthalmic surgery, lasers & imaging retina*. (2015) 46:209-16. doi: 10.3928/23258160-20150213-21

50. Warrow DJ, Lai MM, Patel A, Raevis J, Berinstein DM. Treatment outcomes and spectral-domain optical coherence tomography findings of eyes with symptomatic vitreomacular adhesion treated with intravitreal ocriplasmin. *American journal of ophthalmology*. (2015) 159:20-30.e1. doi: 10.1016/j.ajo.2014.09.015

51. Dyer D, Anderson W, Ellis MP, Breeden R. Extended follow-up of ocriplasmin for vitreomacular traction release. *Investigative Ophthalmology and Visual Science*. (2015) 56:1204.

52. Zhao Y, Itoh Y, Ehlers JP, Srivastava SK, Singh RP, Kaiser PK. 1 year outcomes of intravitreal octriplasmin for symptomatic vitreomacular traction. *Investigative Ophthalmology and Visual Science*. (2015) 56:1230.

53. Coskey A, Brown DM, Hooten C, Kao LK, Wykoff CC, Major JC, et al. Ocriplasmin for vitreomacular adhesion (VMA) in the clinical setting: Rates of VMA release, development of macular holes, and visual outcomes. *Investigative Ophthalmology and Visual Science*. (2014) 55:291.

54. Steinle NC, Quezada C, Nasir M, Pieramici DJ, Castellarin A, See RF, et al. Outer band reflectivity changes on SDOCT following intravitreal ocriplasmin for vitreomacular traction (VMT) and macular holes (MH). *Investigative Ophthalmology and Visual Science*. (2014) 55:296.

55. Roth DB, Feng HL, Modi KK, Fine HF, Wheatley HM. Predictors of success with intravitreal ocriplasmin in the treatment of symptomatic vitreomacular adhesion. *Investigative Ophthalmology and Visual Science*. (2014) 55:298.

56. Singh RP, Li A, Bedi R, Srivastava S, Sears JE, Ehlers JP, et al. Anatomical and visual outcomes following ocriplasmin treatment for symptomatic vitreomacular traction syndrome. *The British journal of ophthalmology*. (2014) 98:356-60. doi: 10.1136/bjophthalmol-2013-304219

57. Kim BT, Schwartz SG, Smiddy WE, Doshi RR, Kovach JL, Berrocal AM, et al. Initial outcomes following intravitreal ocriplasmin for treatment of symptomatic vitreomacular adhesion. *Ophthalmic surgery, lasers & imaging retina*. (2013) 44:334-43. doi: 10.3928/23258160-20130715-05
